# Exploration of Diazaspiro Cores as Piperazine Bioisosteres in the Development of σ2 Receptor Ligands

**DOI:** 10.3390/ijms23158259

**Published:** 2022-07-27

**Authors:** Kuiying Xu, Chia-Ju Hsieh, Ji Youn Lee, Aladdin Riad, Nicholas J. Izzo, Gary Look, Susan Catalano, Robert H. Mach

**Affiliations:** 1 Department of Radiology, Perelman School of Medicine, University of Pennsylvania, Philadelphia, PA 19104, USA; kuxu@pennmedicine.upenn.edu (K.X.); chiahs@pennmedicine.upenn.edu (C.-J.H.); leejiyo@pennmedicine.upenn.edu (J.Y.L.); alriad@pennmedicine.upenn.edu (A.R.); 2 Cognition Therapeutics Inc., Pittsburgh, PA 15203, USA; nizzo@cogrx.com (N.J.I.); glook@cogrx.com (G.L.); scatalano@cogrx.com (S.C.)

**Keywords:** sigma-2 receptors, TMEM97, diazaspiro cores, bioisosteres

## Abstract

A series of σ2R compounds containing benzimidazolone and diazacycloalkane cores was synthesized and evaluated in radioligand binding assays. Replacing the piperazine moiety in a lead compound with diazaspiroalkanes and the fused octahydropyrrolo[3,4-b] pyrrole ring system resulted in a loss in affinity for the σ2R. On the other hand, the bridged 2,5-diazabicyclo[2.2.1]heptane, 1,4-diazepine, and a 3-aminoazetidine analog possessed nanomolar affinities for the σ2R. Computational chemistry studies were also conducted with the recently published crystal structure of the σ2R/TMEM97 and revealed that hydrogen bond interactions with ASP29 and π-stacking interactions with TYR150 were largely responsible for the high binding affinity of small molecules to this protein.

## 1. Introduction

Sigma receptors are a class of proteins that are gaining increased importance in cell biology. Although they were initially thought to be a member of the opioid receptor family, it is now known that they belong to a discrete class of proteins. It is well established that there are two subtypes in sigma receptors, which are termed sigma-1 (σ1R) and sigma-2 (σ2R) receptors [1]. The σ1R has been well characterized; for example, it was first cloned in 1996 [2] and the crystal structure of this protein was reported by Kruse et al. in 2016 [3]. The σ1R is a 223 amino acid trans-membrane protein that is implicated in a range of biological functions, which include InsP3R-dependent calcium influx from the endoplasmic reticulum (ER) to mitochondria, lipid dynamics, mitochondrial-associated membrane stability, regulation of the ER stress response, and modulating channel and GPRC receptor function in the plasma membrane [4]. This unique range of biological functions is associated with the ability of the σ1R to serve as a binding partner in the formation of protein–protein interactions, with numerous “client proteins”.

The characterization of σ2R has been far more elusive. Initial studies relied on in vitro binding studies using [^3^H]DTG in the presence of (+)-pentazocine to mask σ1R sites and largely focused on the density of this protein in various tissues and in cancer cells grown under cell culture conditions [5,6]. The observation that this protein is expressed in high density in a wide variety of cancer cells, and that the density of the σ2R is higher in proliferating versus quiescent breast cancer cells, ushered in an era that focused on the development of σ2R ligands in the treatment and molecular imaging of cancer [7]. It was subsequently observed that antagonists of the σ2R were able to block the neuronal uptake of Aβ oligomers in cultured neurons, which led to the exploration of σ2R ligands as potential drugs for delaying the clinical progression of Alzheimer’s disease (AD) [8]. Other pharmacological activity associated with the σ2R included the treatment of neuropathic pain, alcohol abuse, and cocaine abuse [9,10,11].

In 2011, Xu et al. [12] reported that the σ2R represented a binding site contained within a protein complex that included the progesterone receptor binding component-1 (PGRMC1), an observation that was subsequently challenged [13,14,15]. In 2017, the σ2R was purified and cloned by Kruse et al., who correctly identified this protein as the endoplasmic reticulum (ER)-resident membrane protein TMEM97 [16]. Since TMEM97 is a known protein, the name of the σ2R was subsequently changed to σ2R/TMEM97. The confusion regarding the association between σ2R/TMEM97 and PGRMC1 was clarified by Riad et al., who demonstrated that these two proteins form a trimeric complex with the LDL receptor and regulate the rate of LDL uptake in cancer cells [17,18]. This group subsequently demonstrated that σ2R/TMEM97 and PGRMC1 form a protein complex with LRP receptors, which regulate the uptake of apolipoproteins in neurons. They also demonstrated that Aβ oligomers are taken up into cultured neurons by forming a complex with apolipoproteins and that the rate of uptake was much higher in Aβ oligomers bound to ApoE3, a known risk factor in AD [19]. This observation may provide a partial explanation for the ability of σ2R antagonists to block Aβ oligomer uptake in neurons and their improvement in behavioral outcomes in murine models of AD. A more recent study demonstrated that σ2R antagonists rescue neuronal dysfunction induced by alpha synuclein aggregates isolated from postmortem samples of Parkinson’s disease (PD) brain [20]. These data clearly implicate σ2R/TMEM97 as a potential drug target in delaying the disease progression in both AD and PD.

**Figure 1:**
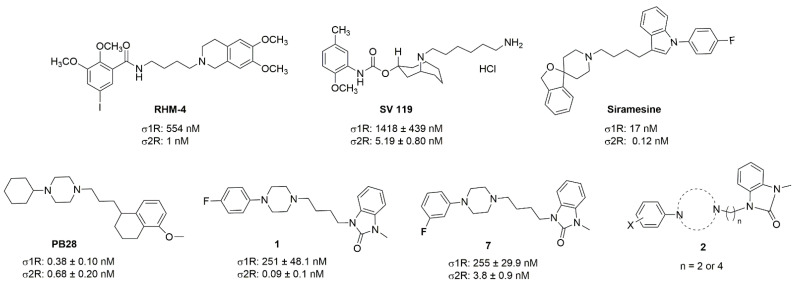
Structures of the σ2R compounds described above.

Although a wide range of structurally diverse compounds has been shown to bind with high affinity to σ2R/TMEM97, only a limited series of compounds display preferential binding to σ2R versus σ1R [7]. Examples of the different classes of compounds displaying this property are shown in Figure 1 and include the 6,7-dimethoxytetrahydroisoquinoline derivatives as **RHM-4** [21], the granatane-related bicyclo-structures represented by **SV119** [22], siramesine and its structural congeners [23,24], as well as the cyclohexylpiperazine analogs represented by **PB28** [25,26,27]. Structurally, they all have two fragments containing an aromatic ring separated by a spacer group containing basic nitrogen, which is a key requirement for high-σ2R affinity. In 2019, McCurdy et al.[28] developed a series of compounds as analogues of **PB28**, containing a piperazine moiety and benzimidazolone (compound **1** in Figure 1), which resulted in a dramatic improvement in the σ2R versus σ1R selectivity of this class of compounds. Our group previously reported that the piperazine of pharmacologically active compounds can be replaced with a di-azaspiroalkanes and other di-azabicyclic fragments and retain higher affinity for their target protein [29,30,31]. Inspired by these reports, we designed a new class of σ2R compounds containing the benzimidazolone scaffold, in which a diazacycloalkane moiety is used as the amine core required for high affinity for the σ2R. The goal of this study was to determine if a similar strategy could be used in the development of compounds having a high affinity for σ2R. The structures of the compounds are represented by generic structure **3** in Figure 1.

## 2. Results

### 2.1. Chemistry

**Scheme 1:**
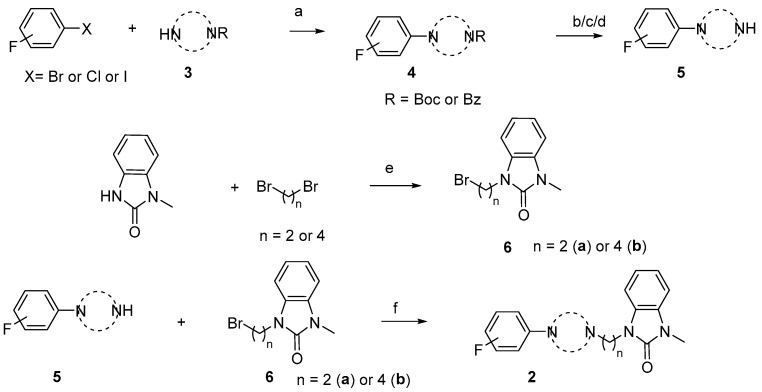
General synthesis of compound **2a-u.** Reagents and conditions: (a) Pd_2_(dba)_3_, RuPhos, aryl halide, NaO-t-Bu, dioxane, 100 °C, 20 min; (b) TFA, DCM, rt, 3 h; (c) 2N HCl in diethyl ether, rt, overnight; (d) H_2_/Pd/C in methanol; (e) NaOH, DMF, rt, overnight; and (f) K_2_CO_3_, DMF, 80 °C.

The synthesis of the targeting compounds began with the N-arylation of the di-azaspiroalkanes or diazacycloalkanes (**3**), as outlined in Scheme 1. Protection of one of the nitrogen atoms in diazaheterocycles, with either a boc- or benzyl group followed by reaction with an aryl halide via a one-pot Pd C-N cross-coupling method [31,32,33], afforded high yields for the protected amines (**4**). Removal of the Boc or benzyl-protecting group provided free amines (**5**) for further coupling. This was accomplished using HCl in diethyl ether or TFA in dichloromethane. It was noted that the 2,6-diazaspiro[3.3]heptane compounds underwent a ring opening upon treatment with HCl and the TFA method was the preferred method for deprotection with this scaffold. Next, the commercially available 1-methyl-1,3-dihydro-2H-benzo[d]imidazol-2-one was alkylated with 1,2-dibromoethane or 1,4-dibromobutane to provide the bromoethyl and bromobutyl derivatives, (**6**) which were then coupled with the amines, (**5**) yielding the desired structures (**2**).

**Scheme 2:**
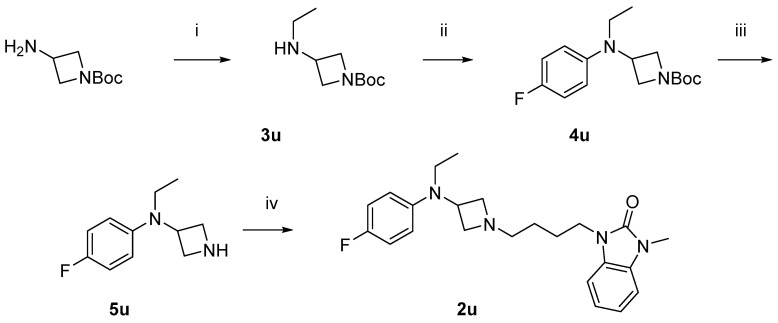
Synthesis of compound **2u.** Reagents and conditions: (i) CH_3_CHO, NaBH(OAc)_3_, MeOH, r.t. overnight; (ii) Pd_2_(dba)_3_, RuPhos, 1-bromo-4-fluorobenzene, NaO-t-Bu, dioxane, 100 °C, 20 min; (iii) HCl in diethyl ether, r.t. overnight; and (iv) **6b**, K_2_CO_3_, DMF, 80 °C.

Scheme 2 shows the synthesis of the compound **2u**. 1-Boc-3-aminoazetidine was ethylated with reductive amination to yield **3u**, which was then arylated with 4-fluorophenyl, giving **4u**. Removal of the Boc-protecting group afforded the free amine **5u,** which was then alkylated to yield the desired product **2u**.

### 2.2. In Vitro Binding Assays

**Figure 2:**
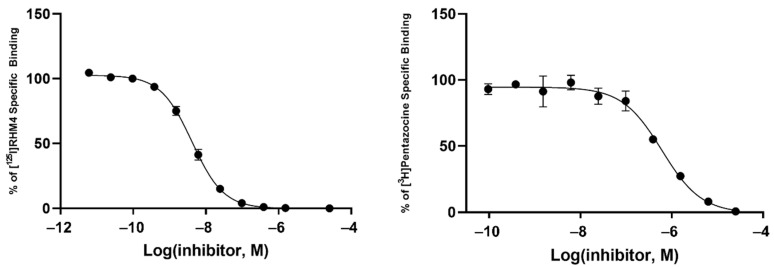
Representative binding curves of compound **2t** for σ2R (**left**) and σ1R (**right**).

Radioligand binding assays were conducted using **[^125^I]RHM-4** with rat liver homogenates for σ2R-binding properties and a σ1R assay using [^3^H]-(+)-pentazocine with Guinea pig brain homogenates. Evaluation of the compounds in the in vitro radioligand binding studies was conducted using a two-step process. An initial screen for σ1 and σ2 receptor affinity was conducted using three different concentrations of the displacer (10 nM, 100 nM, 1μM), as described previously [34]. Compounds displaying ≥ 50% displacement of radioligand binding in σ1 and σ2 assays (Table 1) were chosen for full competition studies using 10 different concentrations of the displacer. A representative competition curve at σ1 and σ2 receptors is shown for compound **2t** in Figure 2. A summary of the full competition assays on hits from the three-point screening assay is provided in Table 2.

Of the 21 analogs (**2a**-**u**) that were synthesized in this study, 6 were identified as “hits” in the three-point screening assay. The full competition data for the six hits revealed that replacement of the piperazine moiety with spirocyclic-diamine resulted in a reduction in affinity for σ2R, but either no change or a modest increase in affinity for the σ1R (Table 1). The homopiperazine analog (**2t**) had the highest affinity for σ2R, followed by the 2,5-diazabicyclo [2.2.1]heptane analog **2r** and the 3-(ethylamino)azetidine analog **2u**. It is also of interest to note that the other bridged analogs (i.e., **2p**, **2q**) and the fused octahydropyrrolo[3,4-b] pyrrole analog **2s** displayed a low affinity for the σ2R.

### 2.3. Computational Chemistry Studies

The recent publication of the crystal structure of σ2R/TMEM97 [35] allowed for the performance of a series of docking and molecular dynamic simulation studies aimed at gaining an understanding of the interactions between the compounds described above and the ligand binding site within this protein. The nitrogen atom adjacent to the carbon chain linker of the piperazine, diazepene, spirocyclic-diamine, 2,5-diazabicyclo[2.2.1]heptane, and 3-(ethylamino)azetidine is predicted to be protonated at physiological pH. The ligand-bound σ2R/TMEM97 crystal structures suggested that the protonated high-affinity ligands form a salt bridge with ASP29 in the σ2R-binding site [35]. Therefore, the binding pose is a salt bridge between the protonated nitrogen of the above amine moieties and ASP29 in docking studies and was considered to determine the estimated binding energy and distance between the protonated nitrogen and ASP29 (Table 3). The estimated binding energies of all the eight compounds were in a range of −10.97 to −9.65 kcal/mol and **7** was observed to have the lowest binding energy. The distances between the protonated nitrogen and ASP29 were between 2.5 and 3.3 Å.

The root mean square distance (RMSD) of all compounds was calculated over 20–100 ns in five copies of the production molecular dynamics simulation (MDS) and used the first time frame (0 ns) as the reference position to evaluate the stability of the ligands in the σ2R-binding pocket. Over the MDS production runs, compound **1,** which had the highest potency in σ2R, showed the least movement and lowest standard deviation of RMSD (RMSD = 2.46 ± 0.11 Å) in the binding pocket (Table 3). Compound **2n**, which had the lowest binding affinity, showed a relatively large amount of motion and the highest standard deviation of RMSD (RMSD = 3.39 ± 2.30 Å) in the binding site.

**Figure 3:**
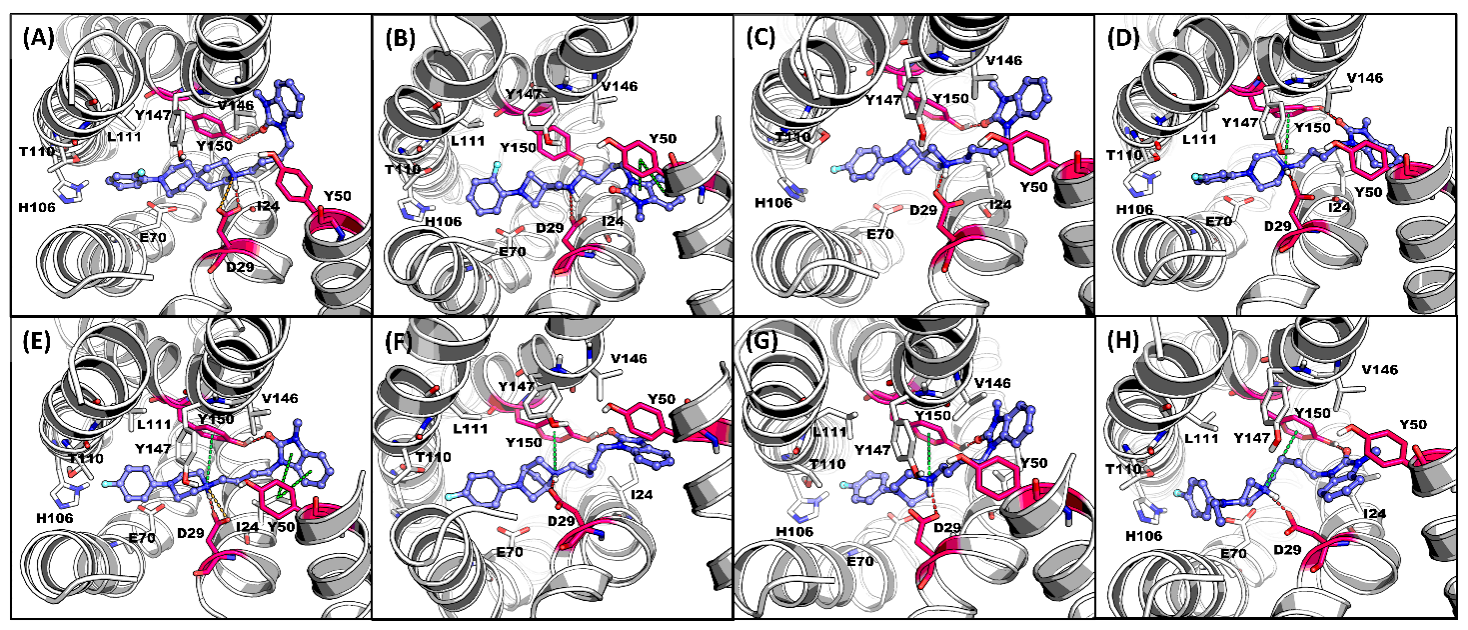
Representative MDS screenshot with the interactive residues in σ2R-binding pocket for (**A**) **2e**, (**B**) **2n**, (**C**) **2o**, (**D**) **7**, (**E**) **1**, (**F**) **2r**, (**G**) **2t**, and (**H**) **2u**. H-Bond: red; salt bridge: orange; π interaction: green. Key interactive residues ASP29, TYR50, and TYR150: red.

An illustration of the binding pose of each compound in the σ2R over the MDS is displayed in Figure 3. All eight selected compounds formed a salt bridge or hydrogen bond between ASP29 and the protonated nitrogen in the ligands. Compound **7**, **1**, **2r**, **2t**, and **2u** formed T-stacking between TYR150 and the protonated nitrogen in the ligands (Figure 3D–H). A cation–π interaction between TYR50 and the benzimidazolinone of the ligands was observed on **2n** and **1** (Figure 3B,E).

**Figure 4:**
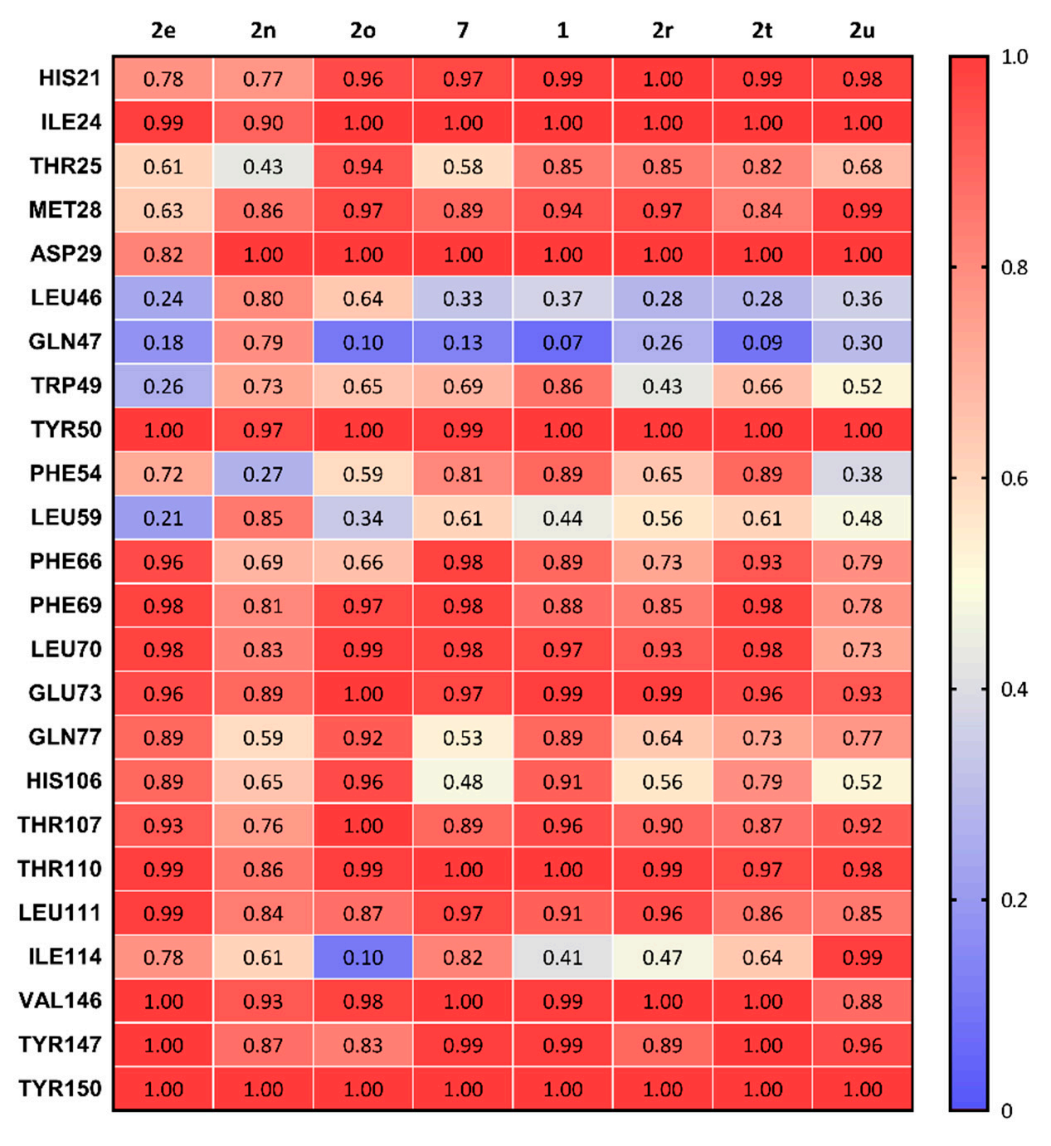
Summary of the frequency of all types of contacts between sigma-2 receptor and 8 compounds.

Summation of the overall frequency of contacts of each ligand to the binding site of σ2R are shown in Figure 4. All eight compounds form stable interactions (frequency of contact > 0.8) with ILE24, ASP29, TYR50, GLU73, THR110, LEU111, VAL146, TYR147, and TYR150. The frequency of contact of **2n** (lowest potency for σ2R) in the main interactive residues was observed as lower overall than other compounds and showed a high interaction with LEU46 and GLN47, two residues located at the entry of the σ2R.

**Figure 5:**
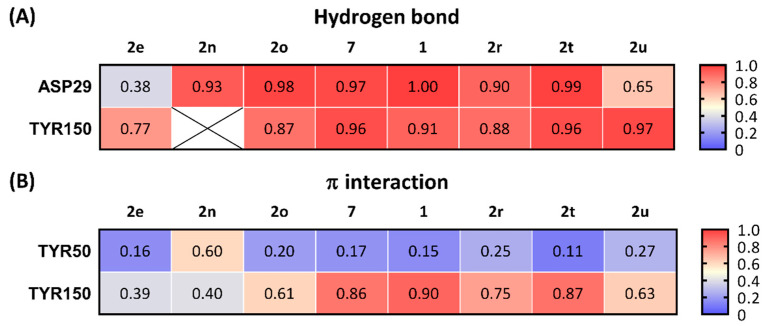
Frequency of contacts for three key interactions between ligands and σ2R. (**A**) Hydrogen bond and (**B**) π interactions. ⊠: No detection of the interactions between ligand and σ2R.

**Figure 6:**
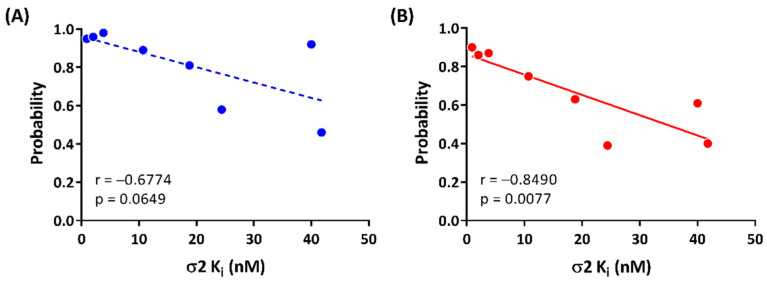
Correlations between σ2R-binding affinities and (**A**) average of frequency of hydrogen bonds for ASP29 and TYR150 and (**B**) frequency of π interaction of TYR150.

All the selected compounds formed a hydrogen bond between ASP29 and the protonated nitrogen in the ligands and TYR150 and the oxygen at the benzimidazolinone of the ligands. Compounds having high potency for σ2R (**1**, **2**, and **2t**; K_i_ < 10 nM) were observed forming stable hydrogen bonds with both ASP29 and TYR150 (frequency of contact > 0.90; Figure 5A). Compounds **2r**, **2u**, and **2e**, which had moderate binding affinities for σ2R (K_i_ = 10–25 nM), showed relatively lower probability to form hydrogen bonds with ASP29 and TYR150 (frequency of contact = 0.38–0.97). There was no detected hydrogen bound formation between **2n** and TYR150. The average of the frequency of forming a hydrogen bond for ASP29 and TYR150 trended towards a positive correlation for σ2R-binding affinity (r = −0.6774, and *p* = 0.0649; Figure 6A).

The benzimidazolinone of each ligand forms π interactions with TYR150 and TYR50 of the σ2R. Compounds **1**, **2**, and **2t** (K_i_ values < 10 nM for σ2R) formed stable π interactions with TYR150 (frequency of contact = 0.86–0.90), whereas compounds with lower potency for σ2R (**2e**, **2n**, **2o**, **2r**, and **2u**; K_i_ = 10–42 nM) were observed as having low to moderate frequency of π interactions (0.39–0.75) with TYR150 (Figure 5B). Compound **2n** was the only ligand that showed a moderate probability of π interactions with TYR50 (frequency of contact = 0.60); the remaining seven compounds showed low frequency of π interactions with TYR50 (frequency of contact <0.3). A significant correlation between the frequency of π interaction formation between TYR150 and the benzimidazolinone of the selected ligands was observed (r = −0.8490, and *p* = 0.0077; Figure 6B).

## 3. Discussion

The goal of this study was to determine the effect of replacing the piperazine moiety of compound **1** with different isosteres, such as spirocyclic and bridged diamine ring systems. Our group previously used this strategy to synthesize compounds targeting either the dopamine D3 receptor or the DNA repair enzyme PARP-1 [29,30]. We were somewhat surprised to see that this substitution generally led to a reduction in affinity for σ2R and an increase in affinity for σ1R. The most potent compound in this series was the homopiperazine analog **2t**, which had an affinity of 4 nM for σ2R and modest selectivity versus σ1R. Another compound displaying good affinity for σ2R and modest selectivity versus σ1R was the bridged analog, **2r**.

Although the SAR studies did not lead to high-affinity σ2R-selective compounds, the range of binding affinities in the compounds reported here provided a good opportunity to conduct molecular modeling studies to evaluate the properties important for interacting with this protein. Our results indicate that hydrogen bond interactions with ASP29 and π stacking interactions with TYR150 are the primary drivers of high affinity for σ2R. These results are currently being used in docking and MDS studies to identify new scaffolds having a high affinity for σ2R.

## 4. Materials and Methods

### 4.1. Chemistry

All reagents and solvents for synthesis were purchased and used without further purification. Structures of synthesized chemicals were identified using ^1^H and ^13^C nuclear magnetic resonance (NMR) spectra and mass spectroscopy. ^1^H and ^13^C NMR were recorded in units relative to deuterated solvent (CDCl_3_) as an internal reference by Bruker DMX 500 MHz NMR instrument (Bruker, Billerica, MA, USA). ^1^H chemical shifts are reported in parts per million (ppm) and measured relative to tetramethylsilane (TMS). Mass spectra were acquired using a 2695 Alliance LC/MS (Milford, MA, USA). Purification of synthesized chemicals was conducted on Biotage Isolera One (Biotage, Salem, NH, USA) with a dual-wavelength UV-VIS detector. A detailed description of the synthesis and characterization of the compounds described in this paper can be found in the Supplementary Materials.

### 4.2. Receptor Binding Assays

For screening, [^3^H]-(+)-pentazocine (~50,000 cpm) or [^125^I]RHM-4 (~200,000 cpm) was mixed with 100 nM of tested compounds. Guinea pig brain homogenates (100 µg) or rat liver homogenates (15 µg) were added to mixture and incubated at 37 °C for 90 min. The mixture was filtered by harvester and washed 3 times using cold washing buffer (10 mM Tris-HCl, 150 mM NaCl). Collected filter was mixed with 3 mL of microscintTM20 and counted 1 min/well using beta counter. To obtain specific binding affinities for sigma binding, tested compound was prepared in concentrations ranging from 10–5 to 10–11 M using assay buffer (50 mM Tris-HCl, 0.1% bovine serum albumin (BSA), pH8) and mixed with [^3^H]-(+)-pentazocine or [^125^I]RHM-4. After that, protein was added and incubated at 37 °C for 90 min. The bound form was filtered by Whatman CF/C filter which was soaked in 1% of polyethyleneimine (PEI) in DW and then filters were washed tree times with cold washing buffer. The non-specific binding was determined in the presence of 10 µM of haloperidol or cold RHM-4. Filter was collected and read by scintillation counting (MicroBeta2 ^®^, PerkinElmer, Beaconsfield, UK) or gamma counter (PerkinElmer, Beaconsfield, UK). The data were analyzed using PRISM 8 (GraphPad Inc., San Diego, CA, USA).

### 4.3. Molecular Docking

In total, 8 selected compounds including 2 references and the 6 hit compounds were used for docking and molecular dynamic studies on sigma-2 receptors. Open Babel v3.1.0 [36] was used for predicting protonated status at physiological pH of each compound. Then, the protonated structures were imported to Chem3D Ultra 15.1 (PerkinElmer Informatics, Inc., Beaconsfield, UK) and minimized using MMFF94 force field calculations for preparation for molecular docking studies. Molecular docking studies were performed via the AutoDock 4.2 [37] plugin on PyMOL (pymol.org) (accessed on 24 July 2022). The X-ray structure of σ2R/TMEM97 (PDB ID 7MFI, resolution 2.81 Å) was obtained from the RCSB Protein Data Bank (https://www.rcsb.org/) (accessed on 24 July 2022). Heteroatoms from water, cholesterol, and other small molecules were removed from the structure. Chain A of the four chains was extracted from the protein structure for further docking and molecular dynamics studies. Then, the protein monomer was imported to H++ web server (http://newbiophysics.cs.vt.edu/H++) (accessed on 24 July 2022) [38,39,40] to protonate the σ2R structure at pH 7.0. A grid box with dimensions of 14.6 × 20 × 12 Å^3^ was applied to the σ2R structure covering binding pocket. The Lamarckian Genetic Algorithm with a maximum of 2,500,000 energy evaluations was used to calculate 100 σ2R–ligand binding poses for each compound. The σ2R–ligand complex that reproduced the crystallographic ligand binding pose and with good docking score was reported for each compound.

### 4.4. Molecular Dynamics Simulation (MDS)

The positioning of proteins in membranes (PPM) web server 3.0 [41] was used to calculate the position of the membranes for σ2R. The preparation of MDS was performed on the CHARMM-GUI web server [42]. The Ligand Reader and Modeler module [43,44] was used to generate the topology and parameter files for 8 compounds. The MDS system with FF19SB force field was built by Bilayer Membrane Builder [45,46]. The protein–ligand complexes obtained from docking studies were aligned to the σ2R model generated from the PPM server and the POPC membrane was placed by the PPM σ2R model. The protein, ligand, and membrane complex was solvated in a TIP3P water box with a volume of 50 × 50 × 104 Å^3^, and then Monte-Carlo sampling was used to add 0.15M NaCl for charge neutralization.

The MDS studies were performed via Amber18 [47] on the high-performance computing (HPC) cluster at Center for Biomedical Image Computing and Analytics at the University of Pennsylvania. The input files of system minimization, 6-step equilibration, and production run for MDS were generated from the last step of Membrane Builder [45,46] on the CHARMM-GUI web server [42]. Periodic boundary conditions were used for the MDS studies. SHAKE algorithm was used to constrain bonds involving hydrogen atoms. An energy minimization of 5000 steps was implemented. Then, the minimized system was heated in a 2-step NVT ensemble with constant volume at 310 K for 125 ps with a time step of 1 fs in each step. The system was then equilibrated in a 4-step NPT ensemble at 310 K and 1 atm for total of 1625 ps (125 ps with 1 fs time step at the first step of NPT ensemble, followed by 500 ps with 2 fs time step at the second to the fourth steps of NPT ensemble). The system minimization and equilibration simulations were performed using pmemd.MPI in Amber18 [47] on 40 CPUs. Five copies of the production simulations were performed for 100 ns with a time step of 2 fs in each copy, using pmemd.cuda Am-ber18 [47] on NVIDIA P100 GPU.

The 20 to 100 ns of each production simulation with a total of 4000 frames (800 frames of each 5 production simulation copies) for each compound were used for further MDS analysis. The interactions between ligand and protein in the production simulations were calculated by using the software BINANA v2.1 [48].

## 5. Conclusions

A series of piperazine isosteres of the known σ2R ligand **1** was synthesized and screened for activity in radioligand binding assays for sigma receptors. Although this effort did not result in the identification of high-affinity ligands for σ2R, the wide range of affinities for this receptor provided data that could be used in computational chemistry studies aimed at determining the properties important for binding to σ2R.

## Figures and Tables

**Table 1:** In vitro screening assay to identify hit compounds.

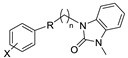				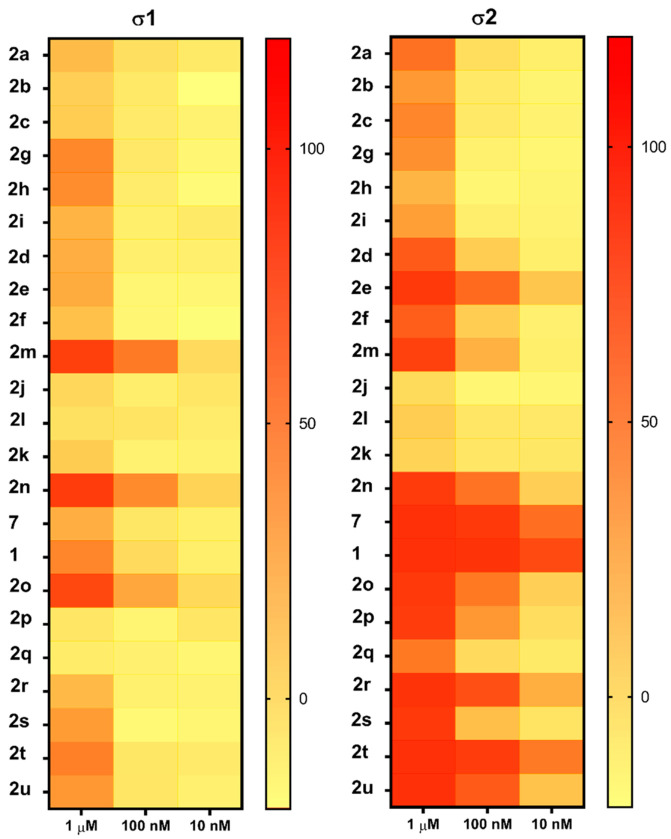
**Cmpd #**	**X**	**R**	**n**	
**2a**	3-F	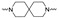	2	
**2b**	2-F	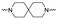	2	
**2c**	4-F	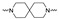	2	
**2d**	3-F	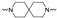	4	
**2e**	2-F	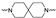	4	
**2f**	4-F	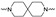	4	
**2g**	3-F	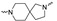	2	
**2h**	2-F	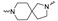	2	
**2i**	4-F	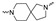	2	
**2j**	3-F	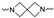	2	
**2k**	2-F	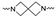	2	
**2l**	4-F	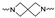	2	
**2m**	3-F	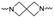	4	
**2n**	2-F	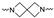	4	
**2o**	4-F	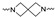	4	
**7** *	3-F	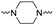	4	
**1** *	4-F	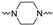	4	
**2p**	4-F	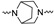	4	
**2q**	4-F	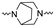	4	
**2r**	4-F	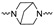	4	
**2s**	4-F	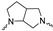	4	
**2t**	4-F	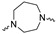	4	
**2u**	4-F	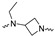	4	* Reference compounds

**Table 2:** Binding affinities of selected compounds for σ2 and σ1 receptors.

	*K*_i_ (nM) ^a^		
Cmpd #	σ2R ^b^	σ1R ^c^	σ1/σ2 Ratio
**2e**	24.4 ± 3.3	659 ± 84.2	27
**2n**	41.8 ± 7.5	20.5 ± 3.3	0.5
**2o**	40.0 ± 3.2	45.9 ± 6.8	1.1
**7** ^d^	2.0 ± 0.6	702 ± 272	351
**1** ^d^	0.9 ± 0.1	251 ± 48.1	279
**2r**	10.7 ± 1.8	712 ± 135	66
**2t**	3.8 ± 0.9	255 ± 29.9	67
**2u**	18.8 ± 1.6	279 ± 30.7	15

^a^ Mean ± SEM, K_i_ values were determined by at least three experiments. ^b^ K_i_ values for σ2R were measured on rat liver homogenates using [^125^I]RHM-4 as the radioligand. ^c^ K_i_ values for σ1R were measured on Guinea pig brain homogenates using [^3^H]-(+)-pentazocine as the radioligand. ^d^ Reference compounds [16].

**Table 3:** Docking and MDS results of selected compounds.

	Docking		MDS
Cmpd #	Distance to ASP29 (Å)	Binding Energy (kcal/mol)	Ligand RMSD (Å)
**2e**	3.3	−10.49	3.50 ± 0.66
**2n**	2.8	−10.36	3.39 ± 2.30
**2o**	3	−9.65	2.62 ± 0.66
**7**	2.5	−10.97	2.69 ± 0.33
**1**	2.8	−10.12	2.46 ± 0.11
**2r**	2.9	−10.43	3.11 ± 0.82
**2t**	3.2	−9.87	2.25 ± 0.32
**2u**	2.6	−10.5	3.24 ± 0.89

## Data Availability

The article contains complete data used to support the findings of this study.

## Supplementary Materials

The following supporting information can be downloaded at: https://www.mdpi.com/article/10.3390/ijms23158259/s1. Reference [49] are cited in the supplementary materials.
