# Supplementary material for: Exploration of Diazaspiro Cores as Piperazine Bioisosteres in the Development of σ2 Receptor Ligands
Source: Int J Mol Sci. 2022 Jul 27;23(15):8259. doi: 10.3390/ijms23158259 (PMC9332756; doi:10.3390/ijms23158259)
Supplement: Supplementary file 1 [file ijms-23-08259-s001.zip › ijms-1813486-supplementary.pdf]

International Journal of *Molecular Sciences*

Article

Exploration of Diazaspiro Cores as Piperazine Bioisosteres in the Development of σ2 Receptor Ligands

Kuiying Xu[1], Chia-Ju Hsieh[1], Ji Youn Lee[1], Aladdin Riad[1], Nicholas J. Izzo[2], Gary Look[2], Susan Catalano[2], and Robert H. Mach[1*]

1 Department of Radiology, Perelman School of Medicine, University of Pennsylvania, Philadelphia, PA, 19104, USA; kuxu@pennmedicine.upenn.edu (K.X.); chiahs@pennmedicine.upenn.edu (C.-J.H.); LeeJiYo@pennmedicine.upenn.edu (J.Y.L.); alriad@PennMedicine.upenn.edu (A.R.); rmach@pennmedicine.upenn.edu (R.H.M.)

2 Cognition Therapeutics Inc., Pittsburgh, PA, 15203, USA; nizzo@cogrx.com (N.J.I.); glook@cogrx.com (G.L.); scatalano@CogRx.com (S.C.)

* Correspondence: rmach@pennmedicine.upenn.edu

Citation: Xu, K.; Hsieh, C.-J.; Lee, J.Y.; Riad, A.; Izzo, N.J.; Look, G.; Catalano, S.; Mach, R.H. Exploration of Diazaspiro Cores as Piperazine Bioisosteres in the Development of σ2 Receptor Ligands. *Int. J. Mol. Sci.* **2022**, *23*, 8259. https://doi.org/10.3390/ijms23158259

Academic Editors: Tangui Maurice and Carmen Abate

Received: 27 June 2022
Accepted: 21 July 2022
Published: 27 July 2022

Publisher's Note: MDPI stays neutral with regard to jurisdictional claims in published maps and institutional affiliations.

Copyright: © 2022 by the authors. Licensee MDPI, Basel, Switzerland. This article is an open access article distributed under the terms and conditions of the Creative Commons Attribution (CC BY) license (https://creativecommons.org/licenses/by/4.0/).

Experimental section

Chemistry

All commercial reagents were purchased and used without further purification. Purification of organic compounds were carried out on a Biotage Isolera One flash chromatography (FC) system with a dual-wavelength UV–vis detector. Compound structures and identity were confirmed by ^{1}H and ^{13}C NMR and mass spectrometry. NMR spectra were taken on a Bruker DMX 500 MHz and a Bruker NEO 400 MHz. Chemical shifts (δ) in the NMR spectra (^{1}H and ^{13}C) were referenced by assigning the residual solvent peaks. Compound purity greater than 95% was determined by LCMS analysis using a 2695 Alliance LCMS.

General Procedure A: Amine (**3**, 1eq), aryl halide (1 eq), $Pd_2(dPa)_3$ (1 mol%), Ruphos (2 mol%) and NaO*t*Bu (4.5 eq) were mixed in a sealed vial and 1.4-dioxane (3 ml) was added. The mixture was kept stirring at 100 °C for 10 min. The mixture was diluted with DCM and filtered through a celite pad. The filtrate was condensed and applied to FC (hexanes/ethyl acetate) yielding the coupled product.

General Procedure B: The Boc protected amine (**4**) was dissolved in DCM (2 ml). HCl in diethyl ether (1 N, 2 ml) was added. The mixture was kept stirring at r.t. overnight. The mixture was neutralized with NH_3/CH_3OH (7N) at 0°C and then condensed. The residue was applied to FC (DCM/MeOH) to yield the product.

General Procedure C: The Boc protected amine was dissolved in a mixture of DCM/TFA and the mixture was kept stirring at r.t. overnight. The mixture was neutralized with NH_3/CH_3OH (7N) at 0°C and then condensed. The residue was applied to FC (DCM/MeOH) to yield the product.

General Procedure D: Amine (**5**, 0.2 mmol) and bromide compound (0.2 mmol) were dissolved in DMF (3 ml), K_2CO_3 (100 mg) was added. The mixture was stirred at 50°C overnight. The mixture was diluted with ethyl acetate (20 ml) wand washed with NaHCO3 aqueous, water and brine. The organic layer was dried over Na_2SO_4 and condensed. The residue was applied to FC (ethyl acetate/MeOH 0-10%) yielding the product.

***tert*-Butyl 3-(ethylamino)azetidine-1-carboxylate (3u)**

1-Boc-3-aminoazetidine (344 mg, 2 mmol) was dissolved in methanol (5ml), acetaldehyde (500 mg) was added, followed by the addition of sodium triacetyloxyboronhydride (500 mg, 2.5 mmol). The mixture was kept stirring at r.t. overnight. The mixture was diluted with DCM and washed with $NaHCO_3$ aq. The organic layer was

dried over Na_2SO_4 and condensed, the residue was applied to FC (Ethyl acetate/methanol 0-10%) yielding **3u** (90 mg, 23%). ^{1}HNMR ($CDCl_3$, 400 MHz): δ 4.03-4.07 (m, 2H), 3.53-3.62 (m, 3H), 2.58 (q, *J* = 7.1 Hz, 2H), 1.40 (s, 9H), 1.08 (t, *J* = 7.1 Hz, 3H). ^{13}C NMR ($CDCl_3$, 100 MHz): 156.26, 79.27, 56.82, 47.68, 41.30, 28.31, 15.22. LC–MS (ESI) m/z: 201.35 [M + H].

***tert*-Butyl 9-(3-Fluorophenyl)-3,9-diazaspiro[5.5]undecane-3-carboxylate (4a)**

The title compound was synthesized with 3,9-diazaspiro[5,5]undecane-3-carboxylic acid *tert*-butyl ester (2 mmol) and 1-chloro-3-fluorobenzene (2 mmol) via General Procedure A. It was obtained as a colorless solid (642 mg, 92%). ^{1}HNMR ($CDCl_3$, 400 MHz): 7.16 (ddd, *J* = 8.2, 8.2, 7.2 Hz, 1H), 6.67 (dd, *J* = 8.4, 2.2 Hz, 2H), 6.58 (ddd, *J* = 12.7, 2.3, 2.3 Hz, 1H), 6.48 (ddd, *J* = 8.2, 8.2, 2.3 Hz, 1H), 3.40 (t, *J* = 5.8 Hz, 4H), 3.17 (t, *J* = 5.8 Hz, 4H), 1.63 (t, *J* = 5.8 Hz, 4H), 1.46-1.49 (m, 13H). ^{13}C NMR ($CDCl_3$, 100 MHz): 163.84 (d, *J* = 242.8 Hz), 154.9, 153.09 (d, *J* = 9.8 Hz), 129.98 (d, *J* = 10.0 Hz), 111.04 (d, *J* = 2.2 Hz), 105.21 (d, *J* = 21.3 Hz), 102.47 (d, *J* = 25.2 Hz), 79.33, 44.37, 39.24, 35.12, 34.92, 29.54, 28.42. LC–MS (ESI) m/z: 349.23 [M + H].

***tert*-Butyl 9-(2-Fluorophenyl)-3,9-diazaspiro[5.5]undecane-3-carboxylate (4b)**

The title compound was synthesized with 3,9-diazaspiro[5,5]undecane-3-carboxylic acid *tert*-butyl ester (2 mmol) and 1-fluoro-2-iodobenzene (2 mmol) via General Procedure A. It was obtained as a colorless solid (544 mg, 78%). ^{1}HNMR ($CDCl_3$, 400 MHz): 6.87-7.05 (m, 4H), 3.40 (t, *J* = 5.8 Hz, 4H), 3.02 (t, *J* = 5.6 Hz, 4H), 1.67 (t, *J* = 5.6 Hz, 4H), 1.45-1.50 (m, 13H). ^{13}C NMR ($CDCl_3$, 100 MHz): 155.66 (d, *J* = 245.7 Hz), 154.92, 140.68 (d, *J* = 8.5 Hz), 124.28 (d, *J* = 3.6 Hz), 122.14 (d, *J* = 7.9 Hz), 118.97 (d, *J* = 3.0 Hz), 115.94 (d, *J* = 20.9 Hz), 79.21, 46.45, 46.42, 39.31, 35.51, 35.33, 29.38, 28.39. LC–MS (ESI) m/z: 349.21 [M + H].

***tert*-Butyl 9-(4-Fluorophenyl)-3,9-diazaspiro[5.5]undecane-3-carboxylate (4c)**

The title compound was synthesized with 3,9-diazaspiro[5,5]undecane-3-carboxylic acid *tert*-butyl ester (2 mmol) and 1-bromo-4-fluorobenzene (2 mmol) via General Procedure A. It was obtained as a colorless solid (640 mg, 92%). %). ^{1}HNMR ($CDCl_3$, 400 MHz): 6.86-6.96 (m, 4H), 3.40 (t, *J* = 5.8 Hz, 4H), 3.07 (t, *J* = 5.7 Hz, 4H), 1.65 (t, *J* = 5.6 Hz, 4H), 1.46-1.49 (m, 13H). ^{13}C NMR ($CDCl_3$, 100 MHz): 156.90 (d, *J* = 238.5 Hz), 154.96, 148.36 (d, *J* = 2.1 Hz), 117.87 (d, *J* = 7.4 Hz), 115.37 (d, *J* = 22.1 Hz), 79.30, 46.05, 39.48, 39.06, 35.31, 29.35, 28.43. LC–MS (ESI) m/z: 349.22 [M + H].

***tert*-Butyl 8-(3-fluorophenyl)-2,8-diazaspiro[4.5]decane-2-carboxylate (4g)**

Compound **4g** was synthesized with 3,8-diazaspiro[4,5]decane-2-carboxylic acid *tert*-butyl ester (2 mmol) and 1-chloro-3-fluorobenzene (2 mmol) via General Procedure A. It was obtained as a colorless solid (594 mg, 88.8%). ^{1}HNMR ($CDCl_3$, 400 MHz): 7.14-7.19 (m, 1H), 6.67 (d, *J* = 7.8 Hz, 2H), 6.59 (d, *J* = 12.5 Hz, 1H), 6.49 (dd, *J* = 8.0, 8.0, 1H), 3.37-3.45 (m, 2H), 3.10-3.26 (m, 6H), 1.67-1.77 (m, 6H), 1.46 (s, 9H). ^{13}C NMR ($CDCl_3$, 100 MHz): 163.82 (d, *J* = 242.9 Hz), 154.68, 152.97 (d, *J* = 9.9 Hz), 130.03 (d, *J* = 10.1 Hz), 111.39, 105.49 (d, *J* = 21.4 Hz), 102.87 (d, *J* = 25.4 Hz), 79.22, 79.14, 55.61, 54.91, 46.55, 46.45, 43.18, 43.88, 40.41, 39.57, 36.22, 35.09, 34.22, 28.50. LC–MS (ESI) m/z: 335.29 [M + H].

***tert*-Butyl 8-(2-fluorophenyl)-2,8-diazaspiro[4.5]decane-2-carboxylate (4h)**

Compound **4h** was synthesized with 3,8-diazaspiro[4,5]decane-2-carboxylic acid *tert*-butyl ester (2 mmol) and 1-fluoro-2-iodobenzene (2 mmol) via General Procedure A. It was obtained as a colorless solid (462 mg, 69.1%). ^{1}HNMR ($CDCl_3$, 400 MHz): 6.90-7.06 (m, 4H), 3.36-3.45 (m, 2H), 3.27 (s, 1H), 3.18 (s, 1H), 3.07-3.12 (m, 2H), 2.92-2.99 (m, 2H), 1.71-1.77 (m, 6H), 1.46(s, 9H). ^{13}C NMR ($CDCl_3$, 100 MHz): 155.70 (d, *J* = 246.1 Hz), 154.69, 140.59 (d, *J* = 8.4 Hz), 124.32, 122.31 (d, *J* = 7.9 Hz), 119.13, 115.97 (d,

J = 20.9 Hz), 79.13, 79.05, 55.79, 54.90, 48.45, 48.40, 44.15, 43.87, 40.26, 39.39, 36.65, 35.36, 34.89, 28.49. LC–MS (ESI) m/z: 335.32 [M + H].

***tert*-Butyl 8-(4-fluorophenyl)-2,8-diazaspiro[4.5]decane-2-carboxylate (4i)**

Compound **4i** was synthesized with 3,8-diazaspiro[4,5]decane-2-carboxylic acid *tert*-butyl ester (2 mmol) and 1-bromo-4-fluorobenzene (2 mmol) via General Procedure A. It was obtained as a colorless solid (515 mg, 77.0%). ^{1}HNMR ($CDCl_3$, 400 MHz): 6.88-6.96 (m, 4H), 3.36-3.44 (m, 2H), 3.25 (s, 1H), 3.12-3.16 (m, 3H), 2.99-3.03 (m, 2H), 1.70-1.76 (m, 6H), 1.46 (m, 9H). ^{13}C NMR ($CDCl_3$, 100 MHz): 157.01 (d, *J* = 239.6 Hz), 154.67, 148.26 (d, *J* = 4.4 Hz), 118.31 (d, *J* = 7.5 Hz), 118.16 (d, *J* = 7.4 Hz), 115.38 (d, *J* = 21.9 Hz), 79.16, 79.07, 55.62, 54.91, 48.23, 48.07, 44.16, 43.86, 40.20, 39.34, 36.30, 35.17, 34.62, 28.48. LC–MS (ESI) m/z: 335.43 [M + H].

***tert*-Butyl 6-(3-fluorophenyl)-2,6-diazaspiro[3.3]heptane-2-carboxylate (4j)**

Compound 4j was synthesized with *tert*-butyl 2,6-diazaspiro[3,3]heptane-2-carboxylate oxalate (1 mmol) and 1-chloro-3-fluorobenzene (1 mmol) via General Procedure A. It was obtained as a colorless solid (287 mg, 98%). ^{1}HNMR ($CDCl_3$, 400 MHz): 7.11-7.16 (m, 1H), 6.42-6.46 (m, 1H), 6.19 (dd, *J* = 8.1, 1.6 Hz, 1H), 6.10-6.14 (m, 1H), 4.08 (s, 4H), 3.96 (s, 4H), 1.44 (s, 9H). ^{13}C NMR ($CDCl_3$, 100 MHz): 163.79 (d, *J* = 243.9 Hz), 155.96, 152.60 (d, *J* = 10.2 Hz), 130.18 (d, *J* = 10.0 Hz), 107.29 (d, *J* = 2.1 Hz), 104.63 (d, *J* = 21.5 Hz), 98.78 (d, *J* = 24.8 Hz), 79.74, 62.10, 59.41, 33.32, 28.31. LC–MS (ESI) m/z: 293.11 [M + H].

***tert*-Butyl 6-(2-fluorophenyl)-2,6-diazaspiro[3.3]heptane-2-carboxylate (4k)**

Compound 4k was synthesized with *tert*-butyl 2,6-diazaspiro[3,3]heptane-2-carboxylate oxalate (1 mmol) and 1-fluoro-2-iodobenzene (1 mmol) via General Procedure A. It was obtained as a colorless solid (270 mg, 92%). ^{1}HNMR ($CDCl_3$, 400 MHz): 6.44-6.48 (m, 1H), 6.69-6.74 (m, 1H), 6.92-7.00 (m, 2H), 4.08 (s, 4H), 4.05 (s, 4H), 1.45 (s, 9H). ^{13}C NMR ($CDCl_3$, 100 MHz): 156.03, 152.52 (d, *J* = 241.5 Hz), , 138.95 (d, *J* = 10.8 Hz), 124.24 (d, *J* = 3.4 Hz), 119.17 (d, *J* = 6.8 Hz), 115.69 (d, *J* = 18.7 Hz), 114.40 (d, *J* = 4.0 Hz), 79.69, 63.42, 63.40, 59.27, 34.17, 34.14, 28.35. LC–MS (ESI) m/z: 393.33 [M + H].

***tert*-Butyl 6-(4-fluorophenyl)-2,6-diazaspiro[3.3]heptane-2-carboxylate (4l)**

Compound **4l** was synthesized with tert-butyl 2,6-diazaspiro[3,3]heptane-2-carboxylate oxalate (1 mmol) and 1-bromo-4-fluorobenzene (1 mmol) via General Procedure A. It was obtained as a colorless solid (280 mg, 96%). ^{1}HNMR ($CDCl_3$, 400 MHz): 6.89-6.92 (m, 2H), 6.36-6.39 (m, 2H), 4.08 (s, 4H), 3.91 (s, 4H), 1.44 (s, 9H). ^{13}C NMR ($CDCl_3$, 100 MHz): 156.28 (d, *J* = 236.1 Hz), 156.00, 147.80 (d, *J* = 1.4 Hz), 115.48 (d, *J* = 22.5 Hz), 112.65 (d, *J* = 7.7 Hz), 79.70, 62.55, 59.42, 33.32, 28.32. LC–MS (ESI) m/z: 293.62 [M + H].

113.00 (d, *J* = 6.5 Hz), 79.67, 57.47, 57.36, 57.23, 57.00, 56.39, 50.92, 50.50, 37.72, 37.30, 28.50, 28.39. LC–MS (ESI) m/z: 294.35 [M + H].

3-Benzyl-9-(4-fluorophenyl)-3,9-diazabicyclo[3.3.1]nonane (4p)

Compound **4p** was synthesized with 3-benzyl-3,9-diazabicyclo[3.3.1]nonane (289 mg, 1 mmol) and 4-fluoro-1-bromobenzene (175 mg, 1 mmol) via General Procedure A as colorless solid (230 mg, 74%). ^{1}HNMR ($CDCl_3$, 400 MHz): 7.35-7.40 (m, 4H), 7.27-7.31 (m, 1H), 6.96 (dd, *J* = 8.7, 8.7 Hz, 2H), 6.75-6.78 (m, 2H), 3.88 (s, 2H), 3.45 (s, 2H), 2.94-3.02 (m, 3H), 2.53 (d, *J* = 12.0 Hz, 2H), 1.97-2.05 (m, 2H), 1.59-1.66 (m, 3H). ^{13}C NMR ($CDCl_3$, 100 MHz): 155.00 (d, *J* = 235.9 Hz), 144.84 (d, *J* = 2.0 Hz), 138.90, 128.65, 128.25, 126.84, 115.71 (d, *J* = 21.7 Hz), 114.77 (d, *J* = 7.1 Hz), 63.53, 57.68, 50.12, 26.39, 20.97. LC–MS (ESI) m/z: 311.39 [M + H].

tert-Butyl 8-(4-fluorophenyl)-3,8-diazabicyclo[3.2.1]octane-3-carboxylate (4q)

Compound **4q** was synthesized with *tert*-butyl 3,8-diazabicyclo[3.2.1]octane-3-carboxylate (212 mg, 1 mmol) and 4-fluoro-1-bromo-benzene (175 mg, 1 mmol) via General Procedure A, yielded 260 mg (85%) as colorless solid. ^{1}HNMR ($CDCl_3$, 400 MHz): 6.94 (dd, *J* = 8.7, 8.6 Hz, 2H), 6.73 (dd, *J* = 9.0, 4.4 Hz, 2H), 4.09 (d, *J* = 19.9 Hz, 2H), 3.73 (d, *J* = 12.6 Hz, 1H), 3.59 (*J* = 12.5 Hz, 1H), 3.28 (d, *J* = 12.6 Hz, 1H), 3.19 (d, *J* = 12.6 Hz, 1H), 1.95-2.03 (m, 2H), 1.79-1.85 (m, 2H), 1.44 (s, 9H). ^{13}C NMR ($CDCl_3$, 100 MHz): 156.07, 155.91 (d, *J* = 237.0 Hz), 143.16 (d, *J* = 1.9 Hz), 116.58 (d, *J* = 7.3 Hz), 116.02 (d, *J* = 22.0 Hz), 79.68, 55.29 55.08, 46.77, 45,53, 28.40, 26.92, 26.83. LC−MS (ESI) m/z: 307.35 [M + H].

***tert*-Butyl 5-(4-fluorophenyl)-2l4,5-diazabicyclo[2.2.1]heptane-2-carboxylate (4r)**

Compound 4r was synthesized from (1*S*, 4*S*)-2-Boc-2,5-diazabicyclo[2.2.1]heptane (198 mg, 1 mmol) and 4-fluoro-1-bromobenzene (130 mg, 1 mmol) via General Procedure A, obtained as colorless solid (250 mg, 85%, mixture of isomers), ^{1}HNMR ($CDCl_3$, 400 MHz): 6.91-6.96 (m, 2H), 6.45-6.48 (m, 2H), 4.61 (s, approx. 0.5H), 4.47 (s, approx. 0.5H), 4.31 (s, 1H), 3.55-3.57 (m, 1H), 3.31-3.49 (m, 2H), 3.15 (d, *J* = 8.5 Hz, approx. 0.5H), 3.05 (d, *J* = 8.5 Hz, approx. 0.5H), 1.87-2.00 (m, 2 H), 1.45 (s, approx. 4.5H), 1.41 (s, approx. 4.5H). ^{13}C NMR ($CDCl_3$, 100 MHz): 154.11, 143.36, 115.75 (d, *J* = 22.4 Hz),

***tert*-Butyl 1-(4-fluorophenyl)hexahydropyrrolo[3,4-b]pyrrole-5(1H)-carboxylate (4s)**

Compound 4s was synthesized with *tert*-butyl hexahydropyrrolo[3,4-b]pyrrole-5(1H)-carboxylate (212 mg, 1 mmol) and 4-fluoro-1-bromobenzene (175 mg, 1 mmol) via General Procedure A as colorless solid (280 mg, 91%). ^{1}HNMR ($CDCl_3$, 400 MHz): 6.92-6.96 (m, 2H), 6.42 (dd, *J* = 9.0, 4.2 Hz, 2H), 4.05-4.15 (m, 1H), 3.50-3.58 (m, 3H), 3.35-3.45 (m, 3H), 3.21-3.29 (m, 1H), 2.90-3.02 (m, 1H), 2.10-2.16 (m, 1H), 1.89-1.95 (m, 1H), 1.43 (s, 9H). ^{13}C NMR ($CDCl_3$, 100 MHz): 155.22 (d, *J* = 234.6 Hz), 154.53, 143.35, 115.57 (d, *J* = 22.2 Hz), 112.69 (d, *J* = 7.1 Hz), 79.41, 67.01, 63.18, 62.29, 60.31, 51.52, 49.78, 49.42, 48.71, 43.78, 41.94, 29.36, 28.39. LC−MS (ESI) m/z: 307.39 [M + H].

tert-Butyl 4-(4-fluorophenyl)-1,4-diazepane-1-carboxylate (4t)

Compound **4t** was synthesized with *tert*-butyl 1,4-diazepane-1-carboxylate (200 mg, 1 mmol) and 4-fluoro-1-bromobenzene (175 mg, 1 mmol) via General Procedure A as colorless solid (350 mg, 89%). NMR data showed a mixture of approx. 5:4 rotmers, ^{13}C NMR reported as peaked. ^{1}HNMR ($CDCl_3$, 400 MHz): 6.91 (dd, *J* = 8.7, 8.6 Hz, 2H), 6.59-6.62 (m, 2H), 3.48-3.56 (m, 6H), 3.31 (t, *J* = 5.7 Hz, 1H), 3.21 (t, *J* = 6.0 Hz, 1H), 1.92-1.95 (m, 2H), 1.42 (s, 5H), 1.36 (s, 4H). ^{13}C NMR ($CDCl_3$, 100 MHz): 156.11, 155.29, 154.96, 153.78, 143.83, 143.65, 115.92, 115.85, 115.70, 115.63, 112.51, 112.38, 112.32, 79.46, 67.03, 60.34, 50.67, 50.43, 49.08, 48.35, 46.23, 46.13, 45.99, 45.58, 28.34, 28.24, 25.22, 24.96. LC−MS (ESI) m/z: 295.30 [M + H].

***tert*-Butyl 3-(ethyl(4-fluorophenyl)amino)azetidine-1-carboxylate (4u)**

Compound **4u** was synthesized with **3u** (200 mg, 1 mmol) and 4-fluorobromobenzene (175 mg, 1 mmol) via General Procedure A as colorless oil (215 mg, KX-07-147 (215 mg, 75%). ^{1}HNMR ($CDCl_3$, 400 MHz): δ 6.92-6.97 (m, 2H), 6.67-6.73 (m, 2H), 4.05-4.15 (m, 3H), 3.80 (br s, 2H), 3.21 (q, *J* = 7.2 Hz, 2H), 1.42 (s, 9H), 0.96 (t, *J* = 7.1 Hz, 3H). ^{13}C NMR ($CDCl_3$, 100 MHz): 157.50 (d, *J* = 240.5 Hz), 156.20, 144.15, 119.91 (d, *J* = 7.3 Hz), 115.64 (d, *J* = 22.2 Hz), 79.51, 53.97, 48.25, 44.86, 28.31, 11.66. LC−MS (ESI) m/z: 295.45 [M + H].

3-(3-Fluorophenyl)-3,9-diazaspiro[5.5]undecane hydrochloride (5a)

Compound **5a** was obtained from **4a** (642 mg, 1.84 mmol) via General Procedure B as a colorless solid (HCl salt) (454 mg, 99%). LC−MS (ESI) m/z: 249.37 [M + H].

3-(2-Fluorophenyl)-3,9-diazaspiro[5.5]undecane hydrochloride (5b)

Compound **5b** was obtained from **4b** (544 mg, 1.84 mmol) via General Procedure B as a colorless solid (HCl salt) (350 mg, 90%). LC−MS (ESI) m/z: 249.27 [M + H].

3-(4-Fluorophenyl)-3,9-diazaspiro[5.5]undecane hydrochloride (5c)

Compound **5c** was obtained from **4c** (600 mg, 1.72 mmol) via General Procedure B as a colorless solid (HCl salt) (480 mg, 98%). LC−MS (ESI) m/z: 249.28 [M + H].

8-(3-Fluorophenyl)-2,8-diazaspiro[4.5]decane (5g)

Compound **5g** was obtained from **4g** (560 mg, 1.68 mmol) via General Procedure B, as colorless solid (356 mg, 90.0%). ^{1}HNMR ($CDCl_3$, 400 MHz): 9.90 (br s, 1H), 7.14-7.20 (m, 1H), 6.65 (d, *J* = 8.4 Hz, 1H), 6.50-6.58 (m, 2H), 3.45 (t, *J* = 7.5 Hz, 2H), 3.17-3.21 (m, 6H), 1.95 (t, *J* = 7.5 Hz, 2H), 1.79-1.84 (m, 4H). ^{13}C NMR ($CDCl_3$, 100 MHz): 163.49 (d, *J* = 243.5 Hz), 152.57 (d, *J* = 9.8 Hz), 130.16 (d, *J* = 10.0 Hz), 111.57 (d, *J* = 2.3 Hz), 106.04 (d, *J* = 21.3 Hz), 103.10 (d, *J* = 25.0 Hz), 53.57, 46.63, 43.56, 41.28, 34.90, 34.10. LC−MS (ESI) m/z: 235.33 [M + H].

8-(2-Fluorophenyl)-2,8-diazaspiro[4.5]decane (5h)

Compound **5h** was obtained from **4h** (440 mg, 1.32 mmol) via General Procedure B, as colorless solid (239 mg, 77.6%). ^{1}HNMR ($CDCl_3$, 400 MHz): 6.91-7.04 (m, 4H), 4.53 (br s, 1H), 3.44 (t, *J* = 7.5 Hz, 2H), 3.19 (s, 2H), 3.00-3.05 (m, 2H), 1.95 (t, *J* = 7.5 Hz, 2H), 1.82-1.86 (m, 4H). ^{13}C NMR ($CDCl_3$, 100 MHz): 155.65 (d, *J* = 245.8 Hz), 140.04 (d, *J* = 8.6 Hz), 124.37 (d, *J* = 3.6 Hz), 122.65 (d, *J* = 8.0 Hz), 119.22 (d, *J* = 2.7 Hz), 116.03 (d, *J* = 20.7 Hz), 53.79, 50.47, 48.31, 48.28, 43.73, 41.01, 35.16, 34.77. LC−MS (ESI) m/z: 235.35 [M + H].

8-(4-Fluorophenyl)-2,8-diazaspiro[4.5]decane (5i)

Compound **5i** was obtained from **4i** (500 mg, 1.50 mmol) via General Procedure B, as colorless solid (328 mg, 93.4%). ^{1}HNMR ($CDCl_3$, 400 MHz):9.91 (br s, 1H), 6.83-6.95 (m, 4H), 3.41-3.46 (m, 2H), 3.16 (s, 2H), 3.04-3.11 (m, 4H), 1.93 (t, *J* = 7.7 Hz, 2H), 1.80-1.84 (m, 4H). ^{13}C NMR ($CDCl_3$, 100 MHz): 157.24 (d, *J* = 239.3 Hz), 147.83 (d, *J* = 2.1 Hz), 118.42 (d, *J* = 7.6 Hz), 115.53 (d, *J* = 22.0 Hz), 53.60, 48.15, 43.59, 41.07, 35.01, 34.53. LC−MS (ESI) m/z: 235.40 [M + H].

2-(3-Fluorophenyl)-2,6-diazaspiro[3.3]heptane (5j)

Compound **5j** was obtained from **4j** (270 mg, 0.92 mmol) via General Procedure C, as colorless solid (140 mg, 79%). ^{1}HNMR (DMSO-d_6, 400 MHz, protonated): 8.97 (br s, 2H), 7.12-7.18 (m, 1H), 6.42-6.46 (m, 1H), 6.23 (d, *J* = 9.1 Hz, 2H), 4.16 (s, 4H), 3.96 (s, 4H). ^{13}C NMR (DMSO-d_6, 100 MHz): 163.24 (d, *J* = 241.1 Hz), 152.91 (d, *J* = 10.7 Hz), 130.38 (d, *J* = 9.2 Hz), 107.68 (d, *J* = 1.9 Hz), 103.62 (d, *J* = 21.4 Hz), 98.41 (d, *J* = 24.7 Hz), 61.09, 54.66, 35.95. LC−MS (ESI) m/z: 193.22 [M + H].

2-(2-Fluorophenyl)-2,6-diazaspiro[3.3]heptane (5k)

Compound **5k** was obtained from **4k** (250 mg, 0.85 mmol) via General Procedure C, as colorless solid (130 mg, 79%). ^{1}HNMR (DMSO-d_6, 400 MHz, protonated): 8.90 (s, 2H), 6.98-7.04 (m, 2H), 6.70-6.73 (m, 1H), 6.51-6.55 (m, 1H), 4.16 (t, *J* = 5.2 Hz, 4H), 4.04 (s, 4H). ^{13}C NMR (DMSO-d_6, 100 MHz): 151.85 (d, *J* = 239.2 Hz), 138.86 (d, *J* = 10.7 Hz), 124.60 (d, *J* = 3.1 Hz), 118.87 (d, *J* = 6.9 Hz), 115.47 (d, *J* = 18.3 Hz), 114.84 (d, *J* = 4.3 Hz), 62.26, 62.24, 54.62, 36.71. LC−MS (ESI) m/z: 193.22 [M + H].

2-(4-Fluorophenyl)-2,6-diazaspiro[3.3]heptane (5l)

Compound **5l** was obtained from **4l** (250 mg, 0.85 mmol) via General Procedure C, as colorless solid (135 mg, 82%). ^{1}HNMR (DMSO-d_6, 400 MHz, protonated): 9.01 (br s, 2H), 6.97-7.01 (m, 2H), 6.40-6.43 (m, 2H), 4.15 (s, 4H), 3.91 (s, 4H). ^{13}C NMR (DMSO-d_6, 100 MHz): 155.47 (d, J = 233.1 Hz), 148.15, 115.29 (d, J = 22.2 Hz), 112.83 (d, J = 7.6 Hz), 61.43, 54.69, 35.93. LC−MS (ESI) m/z: 193.23 [M + H].

9-(4-Fluorophenyl)-3,9-diazabicyclo[3.3.1]nonane (5p)

Compound **5p** (200 mg, 0.65 mmol) was dissolved in methanol (3 ml). 6N HCl (1.5 ml) and pd/C (20%) was added. The mixture was filtered through a celite pad and the filtrate was condensed. The residue was applied to FC (DCM/MeOH 0-10%) yielding KX-07-125 as colorless solid (120 mg, 85%). ^{1}HNMR ($CDCl_3$, 400 MHz): 6.97 (dd, J = 8.0, 8.0 Hz, 2H), 6.73-6.76 (m, 2H), 4.01 (s, 2H), 3.37-3.46 (m, 4H), 2.39-2.43 (m, 1H), 2.05-2.13 (m, 2H), 1.74-1.81 (m, 3H). ^{13}C NMR ($CDCl_3$, 100 MHz): 156.09 (d, J = 236.5 Hz), 143.42, 116.18 (d, J = 21.1 Hz), 115.60 (d, J = 7.3 Hz), 47.35, 45.61, 25.27, 18.52. LC−MS (ESI) m/z: 221.34 [M + H].

8-(4-Fluorophenyl)-3,8-diazabicyclo[3.2.1]octane (5q)

Compound **5q** was synthesized with **4q** (250 mg, 0.85 mmol) via General Procedure B, obtained as brown solid (168 mg, 96%). ^{1}HNMR ($CDCl_3$, 400 MHz): 6.99 (dd, J = 8.5, 8.5 Hz, 2H), 6.70 (dd, J = 8.9, 4.2 Hz, 2H), 4.19 (s, 2H), 3.32 (d, J = 11.7 Hz, 2H), 3.07 (d, J = 12.3 Hz, 1H), 2.22 (s, 4H). ^{13}C NMR ($CDCl_3$, 100 MHz): 156.60 (d, J = 239.1 Hz), 141.62 (d, J = 2.1 Hz), 116.49 (d, J = 22.2 Hz), 116.47 (d, J = 7.4 Hz), 53.82, 44.58, 26.08. LC−MS (ESI) m/z: 207.32 [M + H].

2-(4-Fluorophenyl)-2,5-diazabicyclo[2.2.1]heptane (5r)

Compound **5r** was synthesized from **4r** (300 mg, 0.86 mmol), via General Procedure B, yielded as a colorless oil (190 mg, 98%). ^{1}HNMR ($CDCl_3$, 400 MHz): 10.00 (br s, 1H), 9.49 (br s, 1H), 6.95 (dd, J = 8.6, 8.6 Hz, 2H), 6.47 (dd, J = 8.9, 4.2 Hz, 2H), 4.38 (s, 1H), 4.28 (s, 1H), 3.59 (d, J = 10.4 Hz, 1H), 3.36 (d, J = 10.4 Hz, 1H), 3.24-3.29 (m, 2H), 2.09-2.20 (m, 4H). ^{13}C NMR ($CDCl_3$, 100 MHz): 156.10 (d, J = 236.7 Hz), 141.75, 116.11 (d, J = 22.4 Hz), 113.84 (d, J = 7.2 Hz), 57.71, 55.61, 52.52, 48.79, 36.00. LC−MS (ESI) m/z: 193.25 [M + H].

1-(4-Fluorophenyl)octahydropyrrolo[3,4-b]pyrrole (5s)

Compound 5s was synthesized from **4s** (280 mg, 0.92 mmol) with General Procedure B as a colorless oil (178 mg, 95%). ^{1}HNMR ($CDCl_3$, 400 MHz): 6.95 (dd, J = 8.5, 8.5 Hz, 2H), 6.43 (dd, J = 8.7, 4.0 Hz, 2H), 4.22 (t, J = 5.1 Hz, 1H), 3.60-3.66 (m, 1H), 3.33-3.43 (m, 2H), 3.19-3.26 (m, 2H), 3.12-3.16 (m, 2H), 2.14-2.21 (m, 1H), 2.02-2.10 (m, 1H). ^{13}C NMR ($CDCl_3$, 100 MHz): 155.78 (d, J = 236.2 Hz), 142.80, 115.84 (d, J = 22.1 Hz), 113.52 (d, J = 7.4 Hz), 77.19, 62.61, 50.68, 49.58, 48.91, 42.33, 28.74. LC−MS (ESI) m/z: 207.35 [M + H].

1-(4-fluorophenyl)-1,4-diazepane hydrochloride (5t)

Compound 5t was synthesized starting with **4t** via procedure B. After reaction the solid was filtered and washed with diethyl ether. The product was obtained as the HCl salt without further purification. LC−MS (ESI) m/z: 195.38 [M + H].

***N*-Ethyl-N-(4-fluorophenyl)azetidin-3-amine (5u)**

Compound **5u** was obtained from **4u** (80 mg, 0.27 mmol) via General Procedure B as a colorless solid (50 mg, 95%). ^{1}HNMR ($CDCl_3$, 400 MHz): δ 6.94-7.00 (m, 2H), 6.79-6.83 (m, 2H), 4.55-4.65 (m, 1H), 3.95-4.21 (m, 4H), 3.19-3.24 (m, 2H), 0.93-1.00 (m, 3H). LC−MS (ESI) m/z: 295.45 [M + H].

LC−MS (ESI) m/z: 195.29 [M + H].

1-(2-Bromoethyl)-3-methyl-1,3-dihydro-2H-benzo[d]imidazol-2-one (6a)

1-Methy-2-benzimidazolinone (296 mg, 1 mmol) was dissolved in DMF (10 ml), NaOH (160 mg, 4 mmol) was added. The mixture was kept stirring at r.t. for 1h, and 1,2-dibromoethane (2.6 g, 14 mmol) was added. The mixture was kept stirring at r.t. for 3 h. The mixture was diluted with ethyl acetate (60 ml) and washed with H_2O (60 ml x 2) and brine (60 ml x 2). The organic layer was dried over Na_2SO_4 and condensed. The residue was applied to FC (hexanes/EA 0-60%) yielding **6a** as colorless solid (457 mg, 90%). ^{1}HNMR ($CDCl_3$, 400 MHz): 7.25-7.27 (m, 1H), 7.14-7.16 (m, 1H), 7.03-7.09 (m 2H), 4.24 (t, *J* = 6.3 Hz, 2H), 3.77 (t, *J* = 6.3 Hz, 2H), 3.32 (s, 3H). ^{13}C NMR ($CDCl_3$, 100 MHz): 153.48, 129.60, 128.68, 121.08, 120.95, 108.12, 107.81, 42.13, 30.45, 26.90. LC−MS (ESI) m/z: 255.20 [M + H].

1-(4-Bromobutyl)-3-methyl-1,3-dihydro-2H-benzo[d]imidazol-2-one (6b)

1-Methy-2-benzimidazolinone (444mg, 3 mmol) was dissolved in DMF (15 ml). NaOH (120 mg, 3 mmol) was added. The mixture was kept stirring at r.t for 2h and 1,4-dibromobutane (1.9 mg, 0.9 ml) was added. The mixture was kept stirring at r.t. overnight. The mixture was diluted with ethyl acetate and washed with water (40 ml x 2) and brine (20 ml x 2). The organic layer was dried with Na_2SO_4 and condensed. The residue was applied to FC (hexanes/EA, 0-50%) yielding **6b** as colorless liquid (500 mg, 59%). The characterization is consistent with the reference [1].

1-(2-(9-(3-Fluorophenyl)-3,9-diazaspiro[5.5]undecan-3-yl)ethyl)-3-methyl-1,3-dihydro-2H-benzo[d]imidazol-2-one (2a)

Compound **2a** was synthesized with **5a** (50 mg, 0.2 mmol) and **6a** (51 mg, 0.2 mmol) via General Procedure D, and obtained as a colorless solid (74 mg, 87.7%). ^{1}HNMR ($CDCl_3$, 400 MHz): 7.15 (ddd, *J* = 8.2, 8.2, 7.2 Hz, 1H), 7.03-7.11 (m, 3H), 6.97 (dd, *J* = 5.4, 3.2 Hz, 1H), 6.67 (dd, *J* = 8.4, 1.8 Hz, 1H), 6.57 (d, *J* = 12.7 Hz, 1H), 6.45-6.49 (m, 1H), 4.04 (t, *J* = 7.3 Hz, 2H), 3.41 (s, 3H), 3.15 (t, *J* = 5.6 Hz, 4H), 2.72 (t, *J* = 7.3 Hz, 2H), 2.57 (t, *J* = 5.1 Hz, 4H), 1.56-1.62 (m, 8H). ^{13}C NMR ($CDCl_3$, 100 MHz): 163.81 (d, *J* = 242.6 Hz), 154.23, 153.13 (d, *J* = 9.8 Hz), 130.02, 129.92 (d, *J* = 10.1 Hz) 129.25, 121.14, 121.09, 110.96 (d, *J* = 2.3 Hz), 107.59, 107.37, 105.04 (d, *J* = 21.4 Hz), 102.36 (d, *J* = 25.1 Hz), 55.98, 49.18, 44.36, 38.68, 35.07, 28.99, 27.03. LC−MS (ESI) m/z: 423.52 [M + H].

1-(2-(9-(2-Fluorophenyl)-3,9-diazaspiro[5.5]undecan-3-yl)ethyl)-3-methyl-1,3-dihydro-2H-benzo[d]imidazol-2-one (2b)

Compound **2b** was synthesized with **5b** (50 mg, 0.2 mmol) and **6a** (51 mg, 0.2 mmol) via General Procedure D, and obtained as a colorless solid (63 mg, 75%). ^{1}HNMR ($CDCl_3$, 400 MHz): 6.89-7.10 (m, 8H), 4.03 (t, *J* = 7.3 Hz, 2H), 3.41 (s, 3H), 3.01 (t, *J* = 5.4 Hz, 4H), 2.70 (t, *J* = 7.3 Hz, 2H), 2.55 (t, *J* = 5.2 Hz, 4H), 1.66 (t, *J* = 5.4 Hz, 4H), 1.59 (t, *J* = 5.4 Hz, 4H). ^{13}C NMR ($CDCl_3$, 100 MHz): 155.71 (d, *J* = 245.7 Hz), 154.29, 140.84 (d, *J* = 8.6 Hz), 130.07, 129.35, 124.29 (d, *J* = 3.4 Hz), 122.07 (d, *J* = 8.0 Hz), 121.13, 121.07, 118.99 (d, *J* = 3.0 Hz), 115.96 (d, *J* = 20.9 Hz), 107.59, 107.37, 56.22, 49.37, 46.58, 46.55, 38.93, 35.96, 35.58, 28.96, 27.06. LC−MS (ESI) m/z: 423.53 [M + H].

1-(2-(9-(4-Fluorophenyl)-3,9-diazaspiro[5.5]undecan-3-yl)ethyl)-3-methyl-1,3-dihydro-2H-benzo[d]imidazol-2-one (2c)

Compound 2c was synthesized with **5c** (50 mg, 0.2 mmol) and **6a** (51 mg, 0.2 mmol) via General Procedure D, and obtained as a colorless solid (63 mg, 75%). ^{1}HNMR ($CDCl_3$, 400 MHz): 7.02-7.10 (m, 3H), 6.85-6.98 (m, 5H), 4.03 (t, *J* = 7.3 Hz, 2H), 3.41 (s, 3H), 3.05 (t, *J* = 5.6 Hz, 4H), 2.70 (t, *J* = 7.3 Hz, 2H), 2.54 (t, *J* = 5.2 Hz, 4H), 1.63 (t, *J* = 5.6 Hz, 4H), 1.56 (t, *J* = 5.4 Hz, 4H). ^{13}C NMR ($CDCl_3$, 100 MHz): 156.80 (d, *J* = 238.3 Hz), 154.26, 148.44 (d, *J* = 2.1 Hz), 130.04, 129.33, 121.10, 121.06, 117.77 (d, *J* = 7.4

Hz), 115.31 (d, J = 21.9 Hz), 107.57, 107.37, 56.21, 49.36, 46.05, 38.92, 35.65, 35.40, 28.86, 27.04. LC−MS (ESI) m/z: 423.60 [M + H].

1-(4-(9-(3-Fluorophenyl)-3,9-diazaspiro[5.5]undecan-3-yl)butyl)-3-methyl-1,3-dihydro-2H-benzo[d]imidazol-2-one (2d)

Compound 2d was synthesized with **5a** (50 mg, 0.2 mmol) and **6b** (56.6 mg, 0.2 mmol) via General Procedure D, and obtained as a colorless solid (60 mg, 66.7%). ^{1}HNMR ($CDCl_3$, 400 MHz): 7.14 (ddd, J = 8.0, 8.0, 7.7 Hz, 1H), 7.05-7.10 (m, 2H), 6.94-6.99 (m, 2H), 6.64 (d, J = 8.3 Hz, 1H), 6.56 (d, J = 12.6 Hz, 1H), 6.46 (t, J = 8.2 Hz, 1H), 3.89 (t, J = 7.0 Hz, 2H), 3.40 (s, 3H), 3.13 (t, J = 5.4 Hz, 4H), 2.51-2.54 (m, 6H), 1.74-1.79 (m, 2H), 1.59-1.67 (m, 10H). ^{13}C NMR ($CDCl_3$, 100 MHz): 163.78 (d, J = 242.7 Hz), 154.35, 153.05 (d, J = 9.7 Hz), 129.97, 129.92 (d, J = 9.6 Hz), 129.12, 121.11, 121.05, 110.97 (d, J = 2.2 Hz), 107.48, 107.36, 105.11 (d, J = 21.5 Hz), 102.38 (d, J = 25.1 Hz), 57.57, 48.65, 44.34, 40.55, 35.06, 34.30, 28.96, 27.02, 26.20, 23.13. LC−MS (ESI) m/z: 451.45 [M + H].

1-(4-(9-(2-Fluorophenyl)-3,9-diazaspiro[5.5]undecan-3-yl)butyl)-3-methyl-1,3-dihydro-2H-benzo[d]imidazol-2-one (2e)

Compound **2e** was synthesized with **5b** (50 mg, 0.2 mmol) and **6b** (51 mg, 0.2 mmol) via General Procedure D, and obtained as a colorless solid (56 mg, 62.2%). ^{1}HNMR ($CDCl_3$, 400 MHz): 6.89-7.13 (m, 8H), 3.90 (t, J = 6.8 Hz, 2H), 3.40 (s, 3H), 3.00 (t, J = 5.2 Hz, 4H), 2.56-2.62 (m, 6H), 1.76-1.82 (m, 2H), 1.56-1.70 (m, 10H). ^{13}C NMR ($CDCl_3$, 100 MHz): 155.66 (d, J = 245.8 Hz), 154.41, 140.61 (d, J = 8.6 Hz), 129.95, 129.07, 124.29 (d, J = 3.5 Hz), 122.19 (d, J = 7.9 Hz), 121.20, 121.14, 118.99 (d, J = 3.0 Hz), 115.95 (d, J = 20.8 Hz), 107.52, 107.43, 57.64, 48.86, 46.49, 46.46, 40.45, 35.79, 34.38, 28.86, 27.05, 26.12, 22.98. LC−MS (ESI) m/z: 451.50 [M + H].

1-(4-(9-(4-Fluorophenyl)-3,9-diazaspiro[5.5]undecan-3-yl)butyl)-3-methyl-1,3-dihydro-2H-benzo[d]imidazol-2-one (2f)

Compound **2f** was synthesized with **5c** (50 mg, 0.2 mmol) and **6b** (51 mg, 0.2 mmol) via General Procedure D, and obtained as a colorless solid (47 mg, 52.2%). ^{1}HNMR ($CDCl_3$, 400 MHz): 7.09-7.11 (m, 2H), 6.85-7.01 (m, 6H), 3.91 (t, J = 6.7 Hz, 2H), 3.41 (s, 3H), 3.06 (t, J = 5.4 Hz, 4H), 2.61-2.78 (m, 6H), 1.78-1.84 (m, 2H), 1.64-1.72 (m, 10H). ^{13}C NMR ($CDCl_3$, 100 MHz): 156.97 (d, J = 238.7 Hz), 154.49, 148.25 (d, J = 2.0 Hz), 130.00, 129.06, 121.29, 121.24, 117.93 (d, J = 7.6 Hz), 115.40 (d, J = 22.0 Hz), 107.55, 107.50, 57.44, 48.80, 46.07, 40.30, 33.81, 28.77, 27.10, 26.06, 22.58. LC−MS (ESI) m/z: 451.56 [M + H].

1-(2-(8-(3-Fluorophenyl)-2,8-diazaspiro[4.5]decan-2-yl)ethyl)-3-methyl-1,3-dihydro-2H-benzo[d]imidazol-2-one (2g)

Compound 2g was synthesized with **5g** (47 mg, 0.2 mmol) and **6a** (51 mg, 0.2 mmol) via General Procedure D, and obtained as a colorless solid (48 mg, 58.8%). ^{1}HNMR ($CDCl_3$, 400 MHz): 7.14 (ddd, J = 8.0, 7.8, 7.8 Hz, 1H), 7.02-7.11(m, 3H), 6.96-6.98 (m, 1H), 6.66 (dd, J = 8.4, 1.8 Hz, 1H), 6.58 (d, J = 12.6 Hz, 1H), 6.48 (t, J = 8.2 Hz, 1H), 4.01 (t, J = 7.2 Hz, 2H), 3.41 (s, 3H), 3.11-3.15 (m, 4H), 2.78 (t, J = 7.2 Hz, 2H), 2.69 (t, J = 6.9 Hz, 2H), 2.52 (s, 2H), 1.64-1.69 (m, 6H). ^{13}C NMR ($CDCl_3$, 100 MHz): 163.80 (d, J = 242.7 Hz), 154.29, 153.15 (d, J = 9.7 Hz), 130.01, 129.92 (d, J = 10.0 Hz), 129.35, 121.06, 121.04, 111.30 (d, J = 2.4 Hz), 107.61, 107.36, 105.16 (d, J = 21.4 Hz), 102.69 (d, J = 24.9 Hz), 65.68, 53.97, 53.73, 46.66, 40.18, 39.86, 37.07, 36.27, 27.03. LC−MS (ESI) m/z: 409.56 [M + H].

1-(2-(8-(2-Fluorophenyl)-2,8-diazaspiro[4.5]decan-2-yl)ethyl)-3-methyl-1,3-dihydro-2H-benzo[d]imidazol-2-one (2h)

Compound **2h** was synthesized with **5h** (47 mg, 0.2 mmol) and **6a** (51 mg, 0.2 mmol) via General Procedure D, and obtained as a colorless solid (50 mg, 61.7%). ^{1}HNMR ($CDCl_3$, 400 MHz): 6.89-7.10 (m, 8H), 4.01 (t, J = 7.2 Hz, 2H), 3.41 (s, 3H), 2.98

(t, *J* = 5.4 Hz, 4H), 2.78 (t, *J* = 7.2 Hz, 2H), 2.69 (t, *J* = 6.9 Hz, 2H), 2.53 (s, 2H), 1.65-1.75 (m, 6H). ^{13}C NMR ($CDCl_3$, 100 MHz): 155.71 (d, *J* = 245.7 Hz), 154.28, 140.81 (d, *J* = 8.4 Hz), 130.02, 129.39, 124.26 (d, *J* = 3.5 Hz), 122.06 (d, *J* = 7.9 Hz), 121.05, 121.01, 119.10 (d, *J* = 3.1 Hz), 115.92 (d, *J* = 20.9 Hz), 107.63, 107.32, 65.93, 54.05, 53.78, 48.71, 48.67, 40.25, 39.75, 37.82, 36.69, 27.02. LC−MS (ESI) m/z: 409.53 [M + H].

1-(2-(8-(4-Fluorophenyl)-2,8-diazaspiro[4.5]decan-2-yl)ethyl)-3-methyl-1,3-dihydro-2H-benzo[d]imidazol-2-one (2i)

Compound 2i was synthesized with **5i** (47 mg, 0.2 mmol) and **6a** (51 mg, 0.2 mmol) via General Procedure D, and obtained as a colorless solid (60 mg, 73.4%). ^{1}HNMR ($CDCl_3$, 400 MHz): 7.03-7.09 (m, 3H), 6.85-6.97 (m, 5H), 4.01 (t, *J* = 7.2 Hz, 2H), 3.40 (s, 3H), 3.02 (t, *J* = 5.0 Hz, 4H), 2.78 (t, *J* = 7.2 Hz, 2H), 2.70 (t, *J* = 6.9 Hz, 2H), 2.53 (s, 2H), 1.64-1.72 (m, 6H). ^{13}C NMR ($CDCl_3$, 100 MHz): 156.87 (d, *J* = 238.3 Hz), 154.26, 148.44 (d, *J* = 2.1 Hz), 130.00, 129.34, 121.06, 121.02, 118.10 (d, *J* = 7.5 Hz), 115.29 (d, *J* = 22.1 Hz), 107.60, 107.33, 65.68, 53.97, 53.71, 48.32, 40.12, 39.66, 37.48, 36.37, 27.01. LC−MS (ESI) m/z: 409.49 [M + H].

1-(2-(6-(3-Fluorophenyl)-2,6-diazaspiro[3.3]heptan-2-yl)ethyl)-3-methyl-1,3-dihydro-2H-benzo[d]imidazol-2-one (2j)

Compound **2j** was synthesized with **5j** (58 mg, 0.30 mmol) and **6a** (75 mg, 0.30 mmol) via General Procedure D, obtained as colorless solid (40 mg, 36.7%). ^{1}HNMR ($CDCl_3$, 400 MHz): 7.04-7.11 (m, 4H), 6.96-6.98 (m, 1H), 6.38-6.43 (m, 1H), 6.15 (dd, *J* = 8.1, 2.0 Hz, 1H), 6.06-6.10 (m, 1H), 3.90-3.93 (m, 6H), 3.49 (s, 4H), 3.42 (s, 3H), 2.87 (t, *J* = 6.7 Hz, 2H). ^{13}C NMR ($CDCl_3$, 100 MHz): 163.79 (d, *J* = 243.7), 154.28, 152.81 (d, *J* = 10.3 Hz), 130.09 (d, *J* = 10.2 Hz), 129.99, 129.13, 121.33, 107.53, 107.18 (d, *J* = 2.2 Hz), 104. 27 (d, *J* = 21.5 Hz), 98.61 (d, *J* = 24.8 Hz), 64.44, 61.84, 56.19, 39.31, 34.80, 27.09. LC−MS (ESI) m/z: 367.37 [M + H].

1-(2-(6-(2-Fluorophenyl)-2,6-diazaspiro[3.3]heptan-2-yl)ethyl)-3-methyl-1,3-dihydro-2H-benzo[d]imidazol-2-one (2k)

Compound **2k** was synthesized with 5k (58 mg, 0.30 mmol) and **6a** (75 mg, 0.30 mmol) via General Procedure D, obtained as colorless oil (30 mg, 27.5%). ^{1}HNMR ($CDCl_3$, 400 MHz): 7.07-7.11 (m, 2H), 7.01-7.05 (m, 1H), 6.88-6.98 (m, 3H), 6.65-6.71 (m, 1H), 6.40-6.45 (m, 1H), 3.99 (s, 2H), 3.98 (s, 2H), 3.89 (t, *J* = 6.8 Hz, 4H), 3.45 (s, 4H), 3.41 (s, 3H), 2.83 (t, *J* = 6.8 Hz, 2H). ^{13}C NMR ($CDCl_3$, 100 MHz): 154.25, 152.43 (d, *J* = 241.3 Hz), 139.16 (d, *J* = 10.9 Hz), 129.99, 129.20, 124.15 (d, *J* = 3.1 Hz), 121.25, 121.22, 118.81 (d, *J* = 6.6 Hz), 115.56 (d, *J* = 18.7 Hz), 114.36 (d, *J* = 4.2 Hz), 107.49, 107.47, 64.39, 63.25, 63.23, 56.42, 39.39, 35.61, 35.58, 27.06. LC−MS (ESI) m/z: 367.48 [M + H].

1-(2-(6-(4-Fluorophenyl)-2,6-diazaspiro[3.3]heptan-2-yl)ethyl)-3-methyl-1,3-dihydro-2H-benzo[d]imidazol-2-one (2l)

Compound **2l** was synthesized with **5l** (58 mg, 0.3 mmol) and **6a** (75 mg, 0.3 mmol) via General Procedure D, obtained as colorless solid (50 mg, 45%). ^{1}HNMR ($CDCl_3$, 400 MHz): 7.07-7.11 (m, 2H), 7.01-7.04 (m, 1H), 6.95-6.98 (m, 1H), 6.88 (t, *J* = 8.8 Hz, 2H), 6.32-6.35 (m, 2H), 3.90 (t, *J* = 6.8 Hz, 2H), 3.85 (s, 4H), 3.46 (s, 4H), 3.41 (s, 3H), 2.84 (t, *J* = 6.8 Hz, 2H). ^{13}C NMR ($CDCl_3$, 100 MHz): 156.11 (d, *J* = 235.6 Hz), 154.24, 147.98 (d, *J* = 1.5 Hz), 129.98, 129.15, 121.28, 121.26, 115.37 (d, *J* = 22.5 Hz), 112.55 (d, *J* = 7.6 Hz), 107.49, 64.41, 62.35, 56.27, 39.32, 34.77, 27.06. LC−MS (ESI) m/z: 367.45 [M + H].

1-(4-(6-(3-Fluorophenyl)-2,6-diazaspiro[3.3]heptan-2-yl)butyl)-3-methyl-1,3-dihydro-2H-benzo[d]imidazol-2-one (2m)

Compound **2m** was synthesized with **5j** (58 mg, 0.30 mmol) and **6b** (75 mg, 0.30 mmol) via General Procedure D, obtained as colorless solid (30 mg, 25.4%). ^{1}HNMR

($CDCl_3$, 400 MHz): 7.07-7.15 (m, 3H), 6.97-7.00 (m, 2H), 6.42 (ddd, *J* = 8.4, 8.4, 1.8 Hz, 1H), 6.18 (dd, *J* = 8.1, 1.7 Hz, 1H), 6.10 (dt, *J* = 11.0, 2.2 Hz, 1H), 3.95 (s, 4H), 3.89 (t, *J* = 7.0 Hz, 2H), 3.58 (s, 4H), 3.41 (s, 3H), 2.67 (t, *J* = 7.2 Hz, 2H), 1.75-1.83 (m, 2H), 1.46-1.52 (m, 2H). ^{13}C NMR ($CDCl_3$, 100 MHz): 163.80 (d, *J* = 243.6 Hz), 154.50, 152.71 (d, *J* = 10.4 Hz), 130.14 (d, *J* = 10.1 Hz), 130.01, 129.06, 121.25, 121.23, 107.50, 107.23 (d, *J* = 2.1 Hz), 104.41 (d, *J* = 21.4 Hz), 98.67 (d, *J* = 24.9 Hz), 63.69, 61.81, 57.58, 40.27, 34.34, 27.10, 25.76, 23.57. LC−MS (ESI) m/z: 395.45 [M + H].

1-(4-(6-(2-Fluorophenyl)-2,6-diazaspiro[3.3]heptan-2-yl)butyl)-3-methyl-1,3-dihydro-2H-benzo[d]imidazol-2-one (2n)

Compound **2n** was synthesized with **5k** (57 mg, 0.3 mmol) and **6b** (84 mg, 0.3 mmol) via General Procedure D, obtained as colorless solid (33 mg, 28%). ^{1}HNMR ($CDCl_3$, 400 MHz): 7.09-7.11 (m, 2H), 6.97-6.99 (m, 2H), 6.88-6.93 (m, 2H), 6.35-6.38 (m, 2H), 3.95 (s, 4H), 3.89 (t, *J* = 8.0 Hz, 4H), 3.53 (s, 4H), 3.42 (s, 3H), 2.63 (s, 2H), 1.76-1.81 (m, 2H), 1.46-1.49 (m, 2H). ^{13}C NMR ($CDCl_3$, 100 MHz): 154.51, 147.96, 130.04, 129.11, 121.25, 121.22, 115.57 (d, *J* = 22.4 Hz), 112.66, 112.59, 107.49, 107.53, 107.50, 63.93, 62.37, 56.13, 40.61, 34.42, 27.04, 25.85. LC−MS (ESI) m/z: 395.51 [M + H].

1-(4-(6-(4-Fluorophenyl)-2,6-diazaspiro[3.3]heptan-2-yl)butyl)-3-methyl-1,3-dihydro-2H-benzo[d]imidazol-2-one (2o)

Compound **2o** was synthesized with **5l** (57 mg, 0.3 mmol) and **6b** (84 mg, 0.3 mmol) via General Procedure D, obtained as colorless solid (33 mg, 28%). ^{1}HNMR ($CDCl_3$, 400 MHz): 7.09-7.11 (m, 2H), 6.97-6.99 (m, 2H), 6.88-6.93 (m, 2H), 6.35-6.38 (m, 2H), 3.95 (s, 4H), 3.89 (t, *J* = 8.0 Hz, 4H), 3.53 (s, 4H), 3.42 (s, 3H), 2.63 (s, 2H), 1.76-1.81 (m, 2H), 1.46-1.49 (m, 2H). ^{13}C NMR ($CDCl_3$, 100 MHz): 156.27 (d, *J* = 236.0 Hz), 154.52, 147.71, 129.97, 128.95, 121.31, 121.29, 115.46 (d, *J* = 22.5 Hz), 112.66 (d, *J* = 7.7 Hz), 107.53, 107.50, 63.39, 62.18, 56.91, 50.66, 40.01, 34.21, 27.09, 25.57. LC−MS (ESI) m/z: 395.62 [M + H].

1-(4-(9-(4-Fluorophenyl)-3,9-diazabicyclo[3.3.1]nonan-3-yl)butyl)-3-methyl-1,3-dihydro-2H-benzo[d]imidazol-2-one (2p)

Compound **2p** was synthesized with **5p** (66 mg, 0.3 mmol) and **6b** (84 mg, 0.3 mg) via General Procedure A, obtained as a colorless solid (42 mg, 33%). ^{1}HNMR ($CDCl_3$, 400 MHz): 7.08-7.11 (m, 2H), 6.97-7.02 (m, 2H), 6.89-6.93 (m, 2H), 6.70-6.73 (m, 2H), 3.94 (t, *J* = 7.0 Hz, 2H), 3.82 (s, 2H), 3.43 (s, 3H), 2.88 (d, *J* = 10.6 Hz, 2H), 2.69-2.79 (m, 1H), 2.39 (d, *J* = 10.5 Hz, 2H), 2.26 (t, *J* = 6.6 Hz, 2H), 1.80-1.93 (m, 5H), 1.54-1.57 (m, 4H). ^{13}C NMR ($CDCl_3$, 100 MHz): 154.98 (d, *J* = 235.0 Hz), 154.44, 144.82, 130.05, 129.30, 121.05, 121.01, 115.68 (d, *J* = 21.8 Hz), 114.72 (d, *J* = 7.1 Hz), 107.51, 107.37, 60.35, 57.89, 57.71, 50.05, 44.36, 40.96, 40.17, 33.03, 29.58, 29.49, 27.08, 26.90, 26.40, 26.04, 25,68, 23.83, 21.06, 20.91. LC−MS (ESI) m/z: 423.60 [M + H].

1-(4-(8-(4-Fluorophenyl)-3,8-diazabicyclo[3.2.1]octan-3-yl)butyl)-3-methyl-1,3-dihydro-2H-benzo[d]imidazol-2-one (2q)

Compound **2q** was synthesized with **5q** (62 mg, 0.3 mmol) and **6b** (84 mg, 0.3 mg) via General Procedure A, obtained as a colorless solid (28 mg, 23%). ^{1}HNMR ($CDCl_3$, 400 MHz): 7.07-7.09 (m, 2H), 6.96-6.70 (m, 2H), 6.88-6.92 (m, 2H), 6.67-6.70 (m, 2H), 4.03 (s, 2H), 3.87-3.92 (m, 2H), 3.41 (s, 3H), 2.53-2.55 (m, 2H), 2.39-2.2.42 (m, 2H), 2.24-2.27 (m, 2H), 1.83-1.93 (m, 4H), 1.76-1.79 (m, 2H), 1.47-1.50 (m, 2H). ^{13}C NMR ($CDCl_3$, 100 MHz): 155.52 (d, *J* = 236.4 Hz), 154.29, 143.73, 129.94, 129.20, 121.00, 120.94, 116.35, 116.29, 115.79, 115.71, 115.62, 115.54, 114.68, 114.63, 107.46, 107.41, 107.32, 57.82, 57.66, 56.80, 56.06, 55.06, 49.95, 40.82, 27.72, 27.01, 26.30, 25.97, 25.90, 23.74, 23.67. LC−MS (ESI) m/z: 409.45 [M + H].

1-(4-(5-(4-Fluorophenyl)-2,5-diazabicyclo[2.2.1]heptan-2-yl)butyl)-3-methyl-1,3-dihydro-2H-benzo[d]imidazol-2-one (2r)

Compound **2r** was synthesized with **5r** (57 mg, 0.3 mmol) and **6b** (84 mg, 0.3 mg) via General Procedure A, obtained as a colorless solid (42 mg, 36%). ^{1}HNMR ($CDCl_3$, 400 MHz): 7.05-7.08 (m, 2H), 6.89-6.97 (m, 4H), 6.44-6.47 (m, 2H), 4.16 (s, 1H), 3.84 (t, *J* = 8.0 Hz, 2H), 3.38-3.42 (m, 2H), 3.38 (s, 3H), 3.30-3.32 (m, 1H), 3.09-3.15 (m, 1H), 2.62-2.68 (m, 3H), 2.07-2.13 (m, 1H), 1.95-1.99 (m, 1H), 1.73-1.79 (m, 2H), 1.50-160 (m, 2H). ^{13}C NMR ($CDCl_3$, 100 MHz): 155.32 (d, *J* = 234.8 Hz), 154.39, 143.02, 129.95, 129.08, 121.17, 121.11, 115.72 (d, *J* = 22.2 Hz), 113.24 (d, *J* = 7.3 Hz), 107.49, 107.42, 61.82, 57.20, 56.80, 52.64, 51.62, 40.48, 36.37, 27.04, 25.89, 25.25. LC−MS (ESI) m/z: 395.45 [M + H].

1-(4-(1-(4-Fluorophenyl)hexahydropyrrolo[3,4-b]pyrrol-5(1H)-yl)butyl)-3-methyl-1,3-dihydro-2H-benzo[d]imidazol-2-one (2s)

Compound **2s** was synthesized from **5s** (62 mg, 0.3 mg) and **6b** (84 mg, 0.3 mmol) via General Procedure D as colorless solid (58 mg, 47%). ^{1}HNMR ($CDCl_3$, 400 MHz): 7.02-7.10 (m, 2H), 6.89-6.96 (m, 4H), 6.44-6.48 (m, 2H), 4.02 (t, *J* = 4.0 Hz, 1H), 4.00-4.04 (m, 2H), 3.87 (t, *J* = 7.4 Hz, 2H), 3.37-3.42 (m, 4H), 3.11-3.17 (m, 1H), 2.85-2.93 (m, 1H), 2.60 (d, *J* = 4.0 Hz, 1H), 2.40-2.53 (m, 4H), 2.10-2.20 (m, 1H), 1.82-1.90 (m, 1H), 1.75-1.80 (m, 2H), 1.50-1.58 (m, 2H). ^{13}C NMR ($CDCl_3$, 100 MHz): 155.16 (d, *J* = 234.42 Hz), 154.33, 144.14 (d, *J* = 1.2 Hz), 129.96, 129.18, 121.04, 120.96, 115.40 (d, *J* = 22.1 Hz), 113.28 (d, *J* = 7.2 Hz), 107.49, 107.32, 63.11, 60.16, 59.34, 54.97, 48.79, 42.08, 40.76, 30.35, 27.00, 26.14, 25.42. LC−MS (ESI) m/z: 409.55 [M + H].

1-(4-(4-(4-Fluorophenyl)-1,4-diazepan-1-yl)butyl)-3-methyl-1,3-dihydro-2H-benzo[d]imidazol-2-one (2t)

Compound **2t** was synthesized from **5t** (70 mg, 0.3 mg) and **6b** (84 mg, 0.3 mmol) via General Procedure D as colorless solid (68 mg, 57%). ^{1}HNMR ($CDCl_3$, 400 MHz): δ 7.05-7.08 (m, 2H), 6.95-6.98 (m, 2H), 6.87-6.91 (m, 2H), 6.55-6.58 (m, 2H), 3.87 (t, *J* = 6.9 Hz, 2H), 3.57 (t, *J* = 4.5 Hz, 2H), 3.39-3.42 (m, 5 H), 2.88 (t, *J* = 4.5 Hz, 2H), 2.77 (t, *J* = 5.1 Hz, 2H), 2.68 (t, *J* = 7.5 Hz, 2H), 2.05-2.10 (m, 2H), 1.73-1.80 (m, 2H), 1.63-1.68 (m, 2H). ^{13}C NMR ($CDCl_3$, 100 MHz): 155.03 (d, *J* = Hz), 154.37, 145.54 (d, *J* = 1.7 Hz), 129.92, 129.02, 121.16, 121.12, 115.55 (d, *J* = 21.9 Hz), 112.53 (d, *J* = 7.2 Hz), 107.46, 107.42, 56.87, 55.23, 54.05, 48.02, 47.98, 40.42, 27.03, 26.34, 25.92, 23.28. LC−MS (ESI) m/z: 397.42 [M + H].

1-(4-(3-(Ethyl(4-fluorophenyl)amino)azetidin-1-yl)butyl)-3-methyl-1,3-dihydro-2H-benzo[d]imidazol-2-one (2u)

Compound 2u was synthesized with **5u** and **6b** via General Procedure D as a colorless solid (32 mg, 27%). ^{1}HNMR ($CDCl_3$, 400 MHz): δ 7.07-7.09 (m, 2H), 7.90-7.98 (m, 4H), 6.67-6.70 (m, 2H), 4.07-4.10 (m, 1H), 3.82-3.89 (m, 4H), 3.39 (s, 3H), 3.16 (q, *J* = 7.1 Hz, 2H), 2.98 (t, *J* = 7.5 Hz, 2H), 2.64 (t, *J* = 7.4 Hz, 2H), 1.78 (dt, *J* = 15.0, 7.4 Hz, 2H), 1.48 (dt, *J* = 15.0, 7.4 Hz, 2H), 0.93 (t, *J* = 7.0 Hz, 3H). ^{13}C NMR ($CDCl_3$, 100 MHz): 157.19 (d, *J* = 239.1 Hz), 154.40, 143.94 (d, *J* = 2.3 Hz), 129.99, 129.11, 121.17, 121.11, 119.25 (d, *J* = 7.6 Hz), 115.58 (d, *J* = 22.0 Hz), 107.49, 107.41, 59.70, 58.10, 48.96, 44.52, 40.52, 27.04, 25.89, 23.89, 11.36. LC−MS (ESI) m/z: 397.45 [M + H].

References

1. Mésangeau, C.; Amata, E.; Alsharif, W.; Seminerio, M.J.; Robson, M.J.; Matsumoto, R.R.; Poupaert, J.H.; McCurdy, C.R. Synthesis and pharmacological evaluation of indole-based sigma receptor ligands. *European journal of medicinal chemistry* **2011**, *46*, 5154-5161.
